# Natural genetic variation of the cardiac transcriptome in non-diseased donors and patients with dilated cardiomyopathy

**DOI:** 10.1186/s13059-017-1286-z

**Published:** 2017-09-14

**Authors:** Matthias Heinig, Michiel E. Adriaens, Sebastian Schafer, Hanneke W. M. van Deutekom, Elisabeth M. Lodder, James S. Ware, Valentin Schneider, Leanne E. Felkin, Esther E. Creemers, Benjamin Meder, Hugo A. Katus, Frank Rühle, Monika Stoll, François Cambien, Eric Villard, Philippe Charron, Andras Varro, Nanette H. Bishopric, Alfred L. George, Cristobal dos Remedios, Aida Moreno-Moral, Francesco Pesce, Anja Bauerfeind, Franz Rüschendorf, Carola Rintisch, Enrico Petretto, Paul J. Barton, Stuart A. Cook, Yigal M. Pinto, Connie R. Bezzina, Norbert Hubner

**Affiliations:** 1 Institute of Computational Biology, Helmholtz Zentrum München, München, Germany; 2 Department of Informatics, Technical University of Munich, Munich, Germany; 3 Department of Clinical and Experimental Cardiology, Heart Center, Academic Medical Center, University of Amsterdam, Meibergdreef 9, Amsterdam, 1105AZ The Netherlands; 4 Maastricht Centre for Systems Biology, Maastricht University, Maastricht, The Netherlands; 5 National Heart Research Institute Singapore, National Heart Centre Singapore, 168752 Singapore, Singapore; 6 Division of Cardiovascular & Metabolic Disorders, Duke-National University of Singapore, 169857 Singapore, Singapore; 7 National Heart and Lung Institute, Imperial College London, London, UK; 8 NIHR Cardiovascular Biomedical Research Unit at Royal Brompton and Harefield Hospitals and Imperial College London, London, UK; 9 Medical Research Council (MRC) London Institute of Medical Sciences, Faculty of Medicine, Imperial College London, London, UK; 10 Cardiovascular and Metabolic Sciences, Max-Delbrück-Center for Molecular Medicine (MDC) in the Helmholtz Association, Robert-Rössle-Str. 10, 13125 Berlin, Germany; 11 Institute for Cardiomyopathies Heidelberg & Department of Cardiology, Angiology and Pneumology, University Heidelberg, Heidelberg, Germany; 12 Deutsches Zentrum für Herz-Kreislauf-Forschung, Heidelberg/Mannheim, Germany; 13 Institute of Human Genetics, Genetic Epidemiology, University of Münster, Münster, Germany; 14 Sorbonne Universités, UPMC Univ Paris 06, INSERM UMRS 1166, Team Genomics & Pathophysiology of Cardiovascular Diseases, F-75013 Paris, France; 15 ICAN Institute for Cardiometabolism and Nutrition, F-75013 Paris, France; 16 Université Versailles Saint Quentin, AP-HP, CESP, INSERM U1018, Hôpital Ambroise Paré, Boulogne-Billancourt, France; 17 Department of Pharmacology and Pharmacotherapy, Faculty of Medicine, University of Szeged, Szeged, Hungary; 18 Department of Medicine, University of Miami School of Medicine, Miami, FL USA; 19 Department of Molecular and Cellular Pharmacology, University of Miami School of Medicine, Miami, FL USA; 20 Division of Genetic Medicine, Department of Medicine, Vanderbilt University, Nashville, TN USA; 21 Department of Pharmacology, Northwestern University Feinberg School of Medicine, Chicago, IL USA; 22 Sydney Heart Bank, Department of Anatomy, Bosch Institute, The University of Sydney, Sydney, Australia; 23 Program in Cardiovascular and Metabolic Disorders, Center for Computational Biology, DUKE-NUS Medical School, Singapore, 169857 Singapore; 24 Charité-Universitätsmedizin, Berlin, Germany; 25 Department of Biochemistry, Genetic Epidemiology and Statistical Genetics, CARIM School for Cardiovascular Diseases, Maastricht Center for Systems Biology (MaCSBio), Maastricht University, Maastricht, The Netherlands; 26 Deutsches Zentrum für Herz-Kreislauf-Forschung, Berlin, Germany

**Keywords:** Genetics, Gene expression, eQTL, Dilated cardiomyopathy, Heart

## Abstract

**Background:**

Genetic variation is an important determinant of RNA transcription and splicing, which in turn contributes to variation in human traits, including cardiovascular diseases.

**Results:**

Here we report the first in-depth survey of heart transcriptome variation using RNA-sequencing in 97 patients with dilated cardiomyopathy and 108 non-diseased controls. We reveal extensive differences of gene expression and splicing between dilated cardiomyopathy patients and controls, affecting known as well as novel dilated cardiomyopathy genes. Moreover, we show a widespread effect of genetic variation on the regulation of transcription, isoform usage, and allele-specific expression. Systematic annotation of genome-wide association SNPs identifies 60 functional candidate genes for heart phenotypes, representing 20% of all published heart genome-wide association loci. Focusing on the dilated cardiomyopathy phenotype we found that eQTL variants are also enriched for dilated cardiomyopathy genome-wide association signals in two independent cohorts.

**Conclusions:**

RNA transcription, splicing, and allele-specific expression are each important determinants of the dilated cardiomyopathy phenotype and are controlled by genetic factors. Our results represent a powerful resource for the field of cardiovascular genetics.

**Electronic supplementary material:**

The online version of this article (doi:10.1186/s13059-017-1286-z) contains supplementary material, which is available to authorized users.

## Background

In recent years genome-wide association studies (GWAS) have identified thousands of disease-associated genetic variants. However, the underlying disease-causing molecular mechanisms have remained largely elusive because these variants are located predominantly in the noncoding part of the genome [1]. Many variants have been shown to coincide with regulatory elements residing in the noncoding part of the genome [2, 3]. Large scale analysis of the genetics of intermediate molecular phenotypes, such as gene and transcript expression levels [4–7] and markers of chromatin states [8–11], can be used to identify regulatory variants and to characterize their role in disease [2, 8, 9, 12]. Regulatory elements, and therefore also the effects of variants on the functioning of these elements, can be highly tissue-specific; hence, it is important to investigate the tissue relevant for the disease [2, 3, 7, 13].

Here we characterized global gene expression in the left ventricular myocardium of human hearts to study dilated cardiomyopathy (DCM), a common cause of heart failure ultimately leading to premature death [14]. Myocardial ischemia as well as toxic, metabolic, and immunologic factors [15] can lead to the DCM phenotype. Moreover, genetic susceptibility plays an important role, with at least 23% of DCM cases being familial [16], and more than 50 genes linked to inherited DCM [15]. The most common genetic cause of DCM are truncating mutations in the gene encoding Titin (*TTN*), a giant sarcomeric protein that spans from the A-band to the Z-disc of the sarcomere [17, 18]. These mutations either introduce a premature stop codon [19, 20] or affect alternative splicing of the >100-kb-long messenger RNA [21]. Titin transcript processing is controlled by the DCM-associated splicing factor RBM20 [21–24], which targets a number of additional DCM-associated genes. The myosin heavy chain locus represents a well characterized example of transcriptional regulation of both protein-coding [25] and noncoding DCM-associated genes [26].

In this study we surveyed the cardiac transcriptome of left ventricular tissue of DCM patients and non-diseased donors. These datasets were used to characterize the impact of regulatory variation on gene expression and splicing in the heart, and its relation to the biology of DCM. We analyzed the differences in expression levels between diseased and non-diseased cardiac tissue, identifying 228 differentially expressed genes. Furthermore, we identified regulatory variants impacting gene and exon expression levels. An overlay of our data with published genome-wide association loci for DCM showed that the identified regulatory variants are enriched for SNPs tagging loci associated with DCM risk. Extending our analysis to GWA loci related to heart physiology in general, we were able to identify candidate genes for about 20% of all reported loci.

## Results

### Gene expression differences between DCM and donor samples

We generated a detailed inventory of the heart transcriptome by deep RNA sequencing of heart samples of 97 patients with DCM and 108 non-diseased donors. Selection of the samples and the two study populations are summarized in Additional file 1: Tables S1 and S2. Additional clinical information of the DCM patients is given in Additional file 1: Table S3. On average we generated 168 million mapped paired-end reads per sample (Additional file 1: Tables S4 and S5), providing sufficient depth for detailed characterization of gene expression and alternative splicing. We have quantified gene expression of 57,820 annotated genes (Gencode v19), including protein-coding genes, antisense transcripts and long noncoding RNAs (lncRNAs).

To assess the quality of our data, we first investigated genes with strong association to DCM described in the literature. We investigated gene expression differences between DCM and donor samples for the well-known DCM-related myosin heavy chain genes *MYH6* and *MYH7*. We confirmed that the fraction of the adult *MYH6* transcripts among all myosin heavy chain RNAs is, on average, around 10% in donors and virtually absent in the DCM patients [25] (Additional file 1: Figure S1). Principal component analysis shows that cases and controls are separated along the direction of largest variance (Additional file 1: Figure S2).

**Fig. 1:**
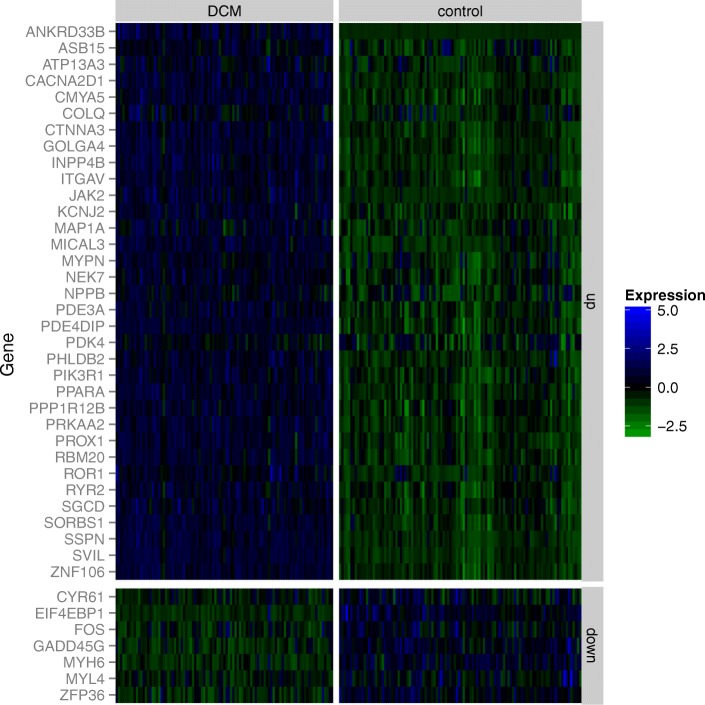
DCM-associated expression of TBX20 targets. Differential expression of human orthologs of TBX20 targets in the mouse heart is shown as a heatmap of gene expression values standardized to mean zero and standard deviation one

We performed a systematic analysis of differential gene expression between DCM cases and non-diseased controls and identified 228 protein-coding genes and 53 noncoding RNAs with significant expression differences and fold changes of at least 20% (Additional files 2 and 3). The top 20 most up- and down-regulated genes are shown in Table 1. Of these, more than half (11/20) have been associated with cardiomyopathy prior to this study while the two most upregulated genes, *NPPA* and *NPPB*, are well established markers of heart failure [27–30]. The latter result confirms the validity of the comparison of relative expression between DCM cases and non-diseased donors. Differentially expressed genes were enriched for Gene Ontology (GO) terms such as structural constituent of muscle (*P* = 4.59E-04), calcium ion binding (*P* = 7.06e-04), regulation of heart contraction (*P* = 2.56e-07), and cardiac tissue development (*P* = 8.77e-05). Among those genes are eight well known DCM-associated genes (Additional file 1: Table S6, reproduced from [20]), which is significantly more than expected by chance (odds ratio (OR) = 7.9, *P* = 2.09e-05).These include *RBM20*, *LAMA2*, and *TBX20*, which were all upregulated in DCM. Differential expression of *TBX20*, an important cardiac transcription factor, is expected to cause expression changes of its target genes. We annotated orthologous human targets of *TBX20* using previously published mouse ChIP-seq data [31] and indeed identified 41 differentially expressed *TBX20* target genes, which are mostly upregulated (OR = 3.3, *P* = 1.0e-9, Fisher’s exact test (FET); Fig. 1).

**Table 1:** Differentially expressed genes with greatest absolute log fold changes

		*P* value	Adjusted *P* value	Log fold change	DCM associated	TBX20 target	CMP associated	Comments
Gene symbol	Description							
NPPA	Natriuretic peptide A	5.61E-09	2.38E-08	0.58	Yes	No	Yes	Natriuretic factor A and B are used as markers of heart failure progression. Natriuretic factor implicated in development and marker of heart failure, also target of T-box factors [27–30]
NPPB	Natriuretic peptide B	1.79E-06	5.57E-06	0.57	Yes	Yes	Yes	See NPPA
TBX20	T-box 20	2.87E-25	3.01E-23	0.49	Yes	No	Yes	TBOX20 has been associated with the pathophysiology of DCM in both animal models and human tissue [82] Furthermore, mutations in TBX20 are associated with familial DCM [83, 84]
MYLK3	Myosin light chain kinase 3	1.07E-21	3.15E-20	0.42	Yes	No	Yes	Associated with stress adaptation and progression to heart failure [85–87]
CLIC5	Chloride intracellular channel 5	2.88E-26	5.40E-24	0.38	No	No	No	CLIC5 is a member of the family of intracellular Ca^2+^ channels, associated with the actin cytoskeletal system. Thus far no link with DCM has been described
TRIM44	Tripartite motif containing 44	4.51E-28	3.73E-25	0.38	No	No	No	Thus far no link with DCM has been described
MAVS	Mitochondrial antiviral signaling protein	5.05E-25	4.67E-23	0.36	No	No	No	Thus far no link with DCM has been described
NPR3	Natriuretic peptide receptor 3	3.68E-23	1.68E-21	0.36	No	No	Yes	NPR3 is the receptor for natriuretic peptides in the heart; it is therefore a candidate for studies into the modulation of NPs in (DCM-related) heart failure [88].
SMCR8	Smith-Magenis syndrome chromosome region, candidate 8	3.66E-28	3.46E-25	0.34	No	No	No	Thus far no link with DCM or the heart has been described
JAK2	Janus kinase 2	2.45E-22	8.67E-21	0.32	No	Yes	Yes	JAK2/STAT3 signaling is, amongst other processes, involved myocardial infarction/reperfusion injury, and hypertrophic remodeling in mice. Thus far no direct link with DCM has been described [89]
TUBA3D	Tubulin alpha 3d	1.66E-08	6.63E-08	-0.26	No	No	No	Thus far no link with DCM or the heart has been described
GADD45B	Growth arrest and DNA damage inducible beta	1.43E-08	5.75E-08	-0.27	No	No	Yes	Changes in expression of *GADD45B* are observed in MI induced HF [90]
DLK1	Delta like non-canonical Notch ligand 1	9.83E-09	4.03E-08	-0.28	No	No	No	Thus far no link with DCM or the heart has been described
TUBA3E	Tubulin alpha 3e	1.17E-10	6.04E-10	-0.30	No	No	No	Thus far no link with DCM or the heart has been described
GADD45G	Growth arrest and DNA damage inducible gamma	2.87E-11	1.58E-10	-0.31	No	Yes	Yes	*Gadd45g* overexpression promotes heart failure and cardiac remodeling after MI; while knockout mice are resistant to heart failure [91]
RASD1	Ras related dexamethasone induced 1	3.32E-07	1.14E-06	-0.32	No	No	No	*RASD1* may be involved in the cardiac release of ANF and BNP upon atrial volume overload in rats [92]. The *RASD1* locus is associated with coronary artery disease in human GWAS [93]
MYL7	Myosin light chain 7	8.29E-10	3.89E-09	-0.33	No	No	No	Thus far no link with DCM has been described
FOS	Fos proto-oncogene, AP-1 transcription factor subunit	5.58E-08	2.09E-07	-0.33	No	Yes	Yes	c-FOS is used as a marker of heart failure [94]
MYH6	Myosin heavy chain 6	2.12E-09	9.50E-09	-0.34	Yes	Yes	Yes	*MYH6* mutations are associated with familial DCM [95]
DHRS7C	Dehydrogenase/reductase 7C	3.31E-09	1.45E-08	-0.39	No	No	Yes	Decrease of *DHRS7C* is observed in mouse models of heart failure and in human cardiac tissue of heart failure patients [96, 97]

### Splicing differences between DCM and donor samples

Alternative splicing is hypothesized to play an important role in the etiology of DCM [21, 24, 32] and other cardiovascular diseases. Here we characterized splicing on the exon level [33–35]. In order to find the differences in exon usage between DCM patients and donors, we used the ‘percentage spliced in’ (PSI) metric that makes use of reads covering the exons as well as the exon–exon junctions. We identified 1212 exons that were significantly different between DCM patients and donors (false discovery rate (FDR) <0.05, ΔPSI >0.1; Additional file 4) corresponding to 899 unique genes. These genes included 11 well known DCM candidate genes (*LDB3*, *LAMA4*, *DTNA*, *TMPO*, *TTN*, *TAZ*, *FLT1*, *DSP*, *SYNE1*, *EYA4*, and *DMD*), which is significantly more than expected by chance (OR = 3.8, FET *P* = 4.4e-04). Differentially spliced genes were enriched for the GO terms MAPK binding (*P* = 6.77E-05), cytoskeleton organization (*P* = 1.07E-07), actin filament organization (*P* = 2.12E-05), Z disc (*P* = 1.10E-03), and I band (*P* = 4.40E-03). We have previously shown that the splicing factor RBM20 is implicated in DCM [21] and directly regulates splicing of *TTN*, *LDB3*, and other *DCM* candidate genes [21, 24]. In this data set the known *RBM20* targets *TTN*, *CAMK2D*, *RTN4*, and paralogs *PDLIM5* and *SORBS2* of the known targets *Pdlim3* and *Sorbs1* were differentially spliced. DCM hearts expressed longer *TTN* isoforms, which is known to cause disease in RBM20-mediated cardiomyopathy [24].

### Genetic effects on the transcriptome

We characterized the impact of naturally occurring genetic variation on the regulation of gene expression and splicing. To this end genotype data were obtained from SNP arrays for all samples. After stringent quality control (see Methods: Additional file 1: Table S7), we imputed variants from the 1000 Genomes project [36]. Imputation quality was assessed using genotype calls obtained from the RNA-seq reads and was high for variants with minor allele frequency (MAF) >10% (Additional file 1: Figure S3). We therefore selected only variants with MAF >10%, resulting in 1,851,329 high confidence imputed variants for quantitative trait locus (QTL) analysis.

*Cis* expression QTL (eQTL) analysis of protein-coding and long noncoding transcripts uncovered widespread genetic effects on gene expression levels. In concordance with earlier studies in cell lines [4–6] and other tissues [7] as well as previous studies in the heart [7], we found eQTL for 17% of protein-coding genes and for 18% of noncoding transcripts (Table 2). In total we identified 188,821 SNPs affecting the transcript levels of 5074 unique genes in the combined expression data of controls and DCM samples adjusted for sex, age, disease status, and additional covariates. We systematically compared our results to eQTL from left-ventricular tissue of the GTEx project [7] (see also Additional file 1: Supplemental notes for a concise summary of all GTEx comparisons), which comprises 190 samples in version 6. For the comparison on the SNP level, we selected the most significant marker for each gene with *cis* eQTL in our study (nominal *P* < 1e-5). Among the top SNPs, 82% were also analyzed in the GTEx study. Of these, 97% had concordant allelic effects (Additional file 1: Figure S4), although only 40% reached the significance threshold in GTEx. The larger number of eQTL detected in our study is most likely due to reduced statistical power caused by a slightly smaller sample size or the use of post-mortem tissues in the GTEx project. Using Storey’s q-value method [37], we estimated that 93% of eQTL are actually shared. Conversely, we have analyzed 18% of the top GTEx SNPs for genes with *cis* eQTL, of which 72% were significant in our study, 97% had concordant allelic effects, and 92% were estimated to be shared. Together these estimates suggest that our study is well replicated by the GTEx study.

**Table 2:** Summary of QTL results

Type	Number tested	Number of significant *cis* QTL	Percentage significant *cis* QTL
Exons	48,119	5,702	11.8
Transcript isoform ratios	19,736	2,874	14.6
Protein-coding	17,323	3,360	19.2
lncRNA	2,887	547	18.5

As *cis*-regulatory variation is dependent on the context, such as the expression or activity of trans-factors (i.e., transcription factors), which might be dramatically altered in the hearts of DCM patients, we studied the presence of DCM/donor specific *cis* eQTL (Additional file 5). Using nested linear models we found 100 DCM-specific and 128 eQTL that were specific for donors. Only three of the specific eQTL SNPs showed evidence of differences in allele frequencies between groups (Armitage trend test, *P* < 0.01). Given the presence of DCM and donor-specific eQTL, we repeated the comparison of eQTL results from each population separately with GTEx (Additional file 1: Figures S5 and S6 and Table S8). We also compared the 100 DCM-specific eQTL with GTEx results and found that only 9.3% were also significant in this non-diseased population.

**Fig. 2:**
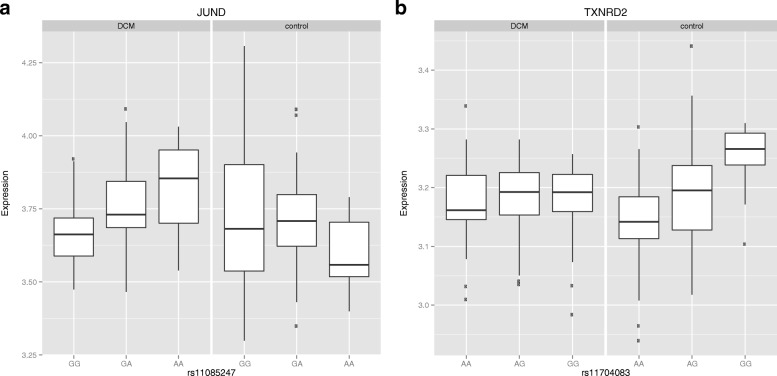
DCM- and control-specific eQTL. Boxplots show examples of eQTL where the genotype only affects expression levels in **a** DCM patients or **b** controls. Expression levels are shown as log transformed normalized read counts. The *x-axis* indicates the genotype of the SNP

We looked for examples of genes with specific *cis* eQTL that have previously been discussed in the context of DCM in the literature. A DCM-specific eQTL was *JUND*, which is specifically expressed during heart development [38]. Higher levels of *JUND* expression are observed in DCM patients. Interestingly, DCM patients carrying the A allele of rs11085247 show even higher expression levels (Fig. 2a). In donors *TXNDRD2* had an eQTL at rs11704083, which was not present in DCM samples (Fig. 2b). Mutations in *TXNDRD2* have been associated with DCM [39] and heart-specific loss of *TXNDRB2* expression leads to a DCM-like phenotype in mice [40].

### Genetic effects on splicing

Since alternative splicing is hypothesized to play an important role in the etiology of DCM [21, 24, 32] and other cardiovascular diseases, we set out to identify splicing QTL (sQTL) using an exon-based model similar to DEXSeq [33]. In addition we also used a gene level test that associates changes of relative transcript isoform abundance with genotypes [41] to identify transcript ratio QTL (trQTL). We found evidence for extensive genetic regulation of splicing, with 11.8% of tested exons and 14.6% of tested genes showing sQTL and trQTL, respectively (Table 2). In both approaches we ruled out confounding by *RBM20* expression by estimating that the upper limit for the fraction of significant *trans* associations of sQTL SNPs to *RBM20* was below 1%. We compared the exon-based (sQTL) and the transcript-based (trQTL) approaches in terms of overlapping genes and overlapping SNPs with significant QTL. The gene level comparison showed that 45% of genes with trQTL were also detected as sQTL and, vice versa, 24% of genes with sQTL were also detected as trQTL. Similar numbers were obtained on the SNP level, with 41 and 26%, respectively. Both comparisons revealed a higher power to detect QTL with the exon-based approach, which is most probably due to the uncertainty in transcript isoform quantification. In comparison to the GTEx study (242 trQTL, ~1.2% of tested genes), we identified over ten times as many trQTL. To investigate the possible factors leading to this increase in detection rate, we matched our data set to the GTEx data set in terms of read depth, sample size and size of the *cis* window (see “Methods”). In this matched analysis we estimated that 1.5% of genes have trQTL (Table 3), which is in accordance with GTEx. This estimate suggests that the increased rate of detection in our study is attributable to the increased sample size, increased *cis* window size, as well as sequencing depth, highlighting the importance of sequencing depth to investigate post-transcriptional regulation (Table 3).

**Table 3:** Effect of read depth, sample size, and covariate adjustment on trQTL detection

*Cis* radius	Reads matched^a^	Samples matched^b^	Adjusted for covariates	Genes tested	Genes with trQTL^c^	Percentage with trQTL^c^
500 kb	Yes	Yes	No	462	19	4.11%
500 kb	Yes	Yes	Yes	457	26	5.69%
500 kb	Yes	No	No	465	43	9.25%
500 kb	Yes	No	Yes	458	58	12.66%
500 kb	No	No	No	19,736	2874	14.56%
500 kb	No	No	Yes	14,586	3588	24.60%
5 kb	Yes	Yes	No	394	6	1.52%
5 kb	Yes	Yes	Yes	377	9	2.39%
5 kb	Yes	No	No	398	32	8.04%
5 kb	Yes	No	Yes	351	33	9.40%
5 kb	No	No	No	16,208	2088	12.88%
5 kb	No	No	Yes	10,304	2469	23.96%

^a^Analysis of chromsome 20 matched to 1.7 million reads corresponding to an estimated total read count of 80 million (GTEx median = 82.1 million)

^b^83 non-diseased samples (GTEx left ventricle, 83)

^c^FDR <0.05

### eQTL and sQTL are overrepresented in known regulatory regions

**Fig. 3:**
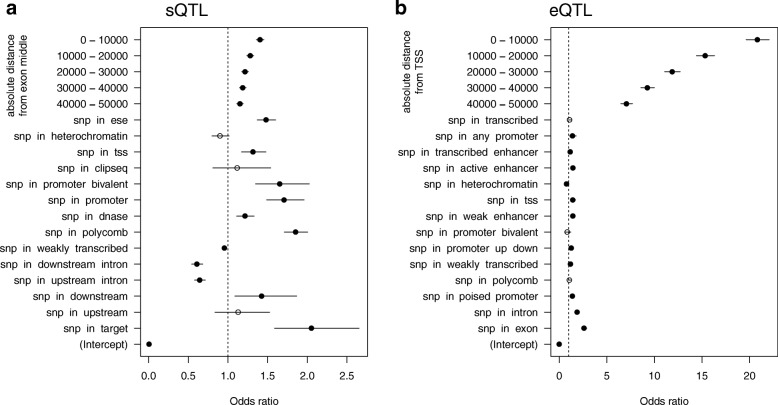
Functional annotation of QTL variants. Enrichment of sQTL (**a**) and (**b**) eQTL in functional categories is shown as estimated odds ratios and 95% confidence intervals of the multiple logistic regression model on the *x-axis* for each annotation category on the *y-axis*. Odds ratios greater than 1 indicate an enrichment of QTL variants in the given functional elements, while odds ratios less than 1 indicate a depletion. Significant odds ratios are shown as *filled circles* (*P* < 0.05)

We functionally annotated the genetic variants that affect gene expression and splicing. eQTL and sQTL have previously been shown to frequently reside in *cis* regulatory elements [6, 7, 42, 43]. We have annotated variants with features based on transcript annotation, position of the variant relative to its target, and an epigenome annotation specific for the left ventricle of the heart from the Epigenomics Roadmap project [44] based on the ChromHMM [45] segmentations of histone modification ChIP-seq data. As described previously [6, 42, 43], we found a strong enrichment of QTL variants around the transcription start site (TSS) for eQTL (Additional file 1: Figure S7a) and around the target exon for sQTL (Additional file 1: Figure S7b). Figure 3 shows that eQTL are enriched in TSSs, exons, and introns even when adjusting for distance effects. Moreover, there was also enrichment in more distant elements such as enhancers, whereas heterochromatin regions were depleted of eQTL. sQTL showed the strongest enrichment when they were located directly within the target exon but also the downstream neighboring exon. In contrast, SNPs in neighboring introns were depleted for sQTL. In addition to the enrichment within the target exon, SNPs that were located in exonic splice enhancer sequences show an enrichment for sQTL, corroborating the important role of these *cis* regulatory sequences for splicing [46]. We also found enrichment of annotations related to transcriptional regulation, such as promoters and DNAse hypersensitivity sites as well as polycomb-associated regions (Fig. 3).

### Genes with allelic imbalance differences are enriched for DCM-related processes

**Fig. 4:**
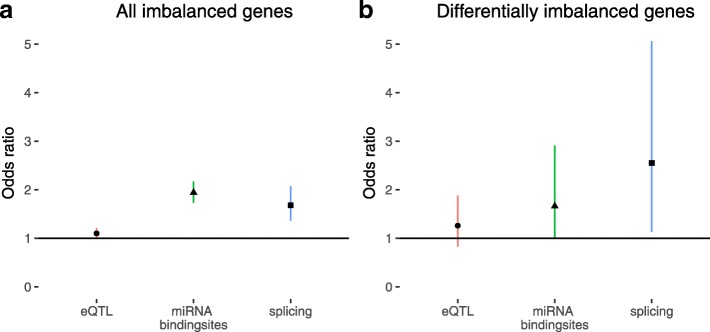
Enrichment for significant eQTLs, miRNA interference, and significant differential splicing in genes with allele-specific expression. Odds ratios with 95% confidence intervals for enrichment are given. **a** All genes with allele-specific expression in at least one individual. Significant enrichment for significant eQTLs, differential splicing, and presence of miRNA binding sites was observed. **b** All genes with differential allele-specific expression between DCM and non-diseased controls with alternative/reference allele frequency difference >0.10. Significant enrichment for differential splicing and presence of miRNA binding sites was observed, with suggestive enrichment for significant eQTLs

Allele specific expression (ASE) is an additional mechanism for naturally occurring variation to affect gene expression. To assess ASE, reference-alternative allele ratios were determined in the aligned RNA-seq reads for all heterozygote sites in each individual, and their deviation from the expected 50:50 ratio was used as a measure of allelic imbalance. Only sites passing strict quality criteria were considered (see “Methods” section) and we observed an overrepresentation of sites located in the 3′ UTR, likely caused by increased sequencing depth due to poly(A) selection for the RNA-seq analysis (Additional file 1: Figure S8). Although this commonly observed technical bias in RNA-seq analysis leads to variants within the 3′ UTR being detected with greater coverage and quality, these variants are used to detect imbalance on the gene level irrespective of relative location. Using this approach, we identified 6499 sites with allelic imbalance (Additional file 6) in at least one individual, corresponding to 3307 genes. Enrichment analysis of these genes revealed enrichments for significant eQTL effects (OR = 1.10, *P* < 0.05, FET) and differential splicing (OR = 1.68, *P* < 2e-06, FET), in addition to presence of miRNA binding sites (OR = 1.94, *P* < 2.2e-16, FET) (Fig. 4a). Enrichment tests for localization are not affected by the 3′ UTR bias, as the background set of all tested variants is also enriched in the 3′ UTR.

We next looked at consistent effects across multiple individuals. Out of all imbalanced genes, 1582 showed a difference larger than 0.10 between alternative and reference allele frequencies consistently across imbalanced individuals. The remaining 1725 genes demonstrate larger inter-individual differences in allelic imbalance, where at the same site either the alternative or reference allele was overexpressed in different individuals, leading to an approximate 50:50 ratio when averaged across individuals. Allelic imbalance observed for these latter sites could be the result of parental imprinting, although overlap with known imprinted genes was only small (*n* = 20).

Next, we compared ASE between the DCM cases and donors. We observed significant differences in the relative number of imbalanced individuals at 448 shared sites. Out of all differential ASE sites, 133 showed a difference between alternative and reference allele ratio larger than 0.10. These sites are located in 132 unique genes (Additional file 7), which were significantly enriched for differential splicing (OR = 2.55, *P* < 0.05, FET) and presence of miRNA binding sites (OR = 1.66, *P* < 0.05, FET) (Fig. 4b). Next, we assessed the biological function of the differentially imbalanced genes. One of the top ten differentially imbalanced genes is *FSTL1* (DCM, 24 out of 42 heterozygotes imbalanced; donors, 10 out of 44 heterozygotes imbalanced; alternative/reference ratio = 40:60; *P* < 0.05, FET). Apart from showing a strong difference in imbalance between DCM cases and donors, *FSTL1* is a known cardioprotective gene, acting as an autocrine/paracrine regulatory factor that antagonizes myocyte hypertrophic growth and the loss of ventricular performance in response to pressure overload [47], and shown to be able to prevent myocardial ischemia/reperfusion injury by inhibiting apoptosis and inflammatory response [48]. The only known DCM-related gene that is differentially imbalanced between DCM cases and donors is *TTN*, but sample size for the heterozygous variant used in the ASE analysis is very low (DCM, 4 out 9 imbalanced; donors, 12 out of 13 imbalanced; alternative/reference ratio = 38:62; *P* < 0.05, FET). Extending on this beyond differential imbalance, we did not observe any consistent strong allele-specific expression effects in *TTN* across all samples. GO enrichment analysis revealed biological processes known to be implicated in DCM [20], including heart development, actin filament-related processes, muscle development and mitochondrial processes (Additional file 1: Table S9). Furthermore, we observed enrichment for genes involved in cytoskeletal protein binding, as well as external matrix and laminin binding, pointing to genes involved in maintaining structural stability on both the cellular and tissue level. Laminins are pivotal for the maintenance and survival of tissues and defects in laminins are known to lead to forms of muscular dystrophy [49, 50]. Together, these results suggest that in DCM hearts, overexpression of specific alleles of genes involved in processes known to play important roles in establishing the DCM phenotype occurs partly through differential splicing and partly through miRNA interference.

### sQTL and eQTL are enriched for DCM variants

**Fig. 5:**
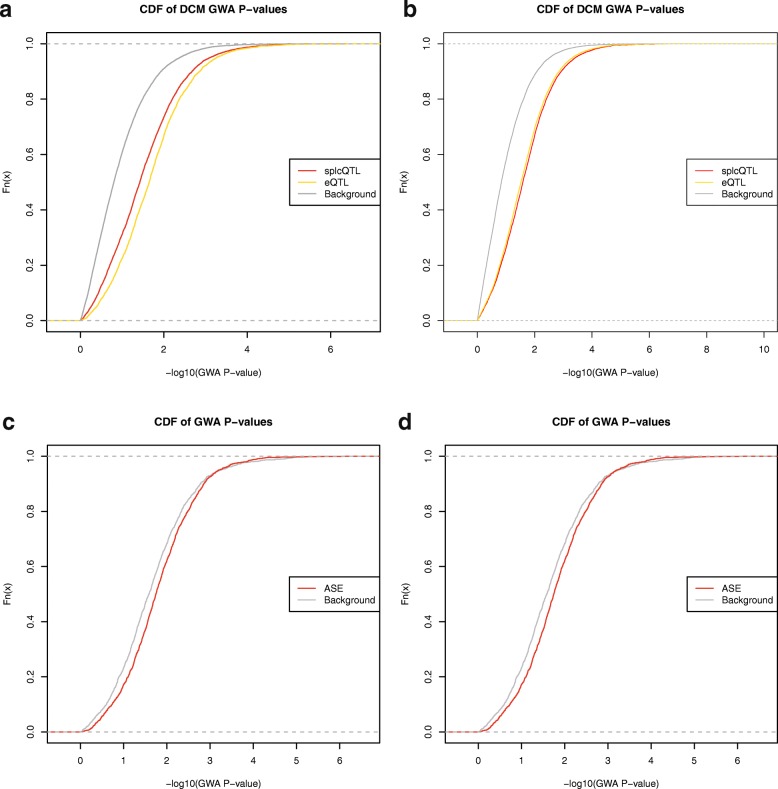
Enrichment of QTL and ASE variants for DCM GWAs. Cumulative density function (CDF) plots for DCM GWA *P* values for LD blocks that have sQTL (*red*) and eQTL (*yellow*) compared to the background set of all tested LD blocks using GWA data from a German DCM population (**a**) and a European DCM population (**b**). Similarly, CDF plots of DCM GWA *P* values for LD blocks with ASE variants (*red*) are compared to the background set of all LD blocks with coding SNPs tested for ASE (*grey*) for a German DCM population (**c**) and a European DCM population (**d**)

We analyzed genome-wide association (GWA) data from two studies that looked for loci involved in DCM in a German population [51] and a set of European populations [52]. We investigated whether the transcriptome altering QTL variants (not specific for DCM or controls) we have identified are enriched for DCM-GWA association signals. Since the GWA studies and eQTL studies were carried out on different genotyping platforms, we defined linkage disequilibrium (LD) blocks [53] with Rsq >0.6 from 1000 Genomes data and tested whether the distributions of association *P* values of LD blocks with and without QTL differ [54]. We found a highly significant enrichment (Fig. 5; *P* < 2.2e-16) of small GWA *P* values for LD blocks with a sQTL in the German DCM GWAS (909 cases versus 2120 controls), which was subsequently replicated (*P* < 2.2e-16) in the international DCM GWAS (1179 cases versus 1108 controls). Similarly, LD blocks with eQTL were also enriched for small GWA *P* values in both GWAS (Fig. 5; *P* < 2.2e-16). A similar comparison of LD blocks with ASE variants to the background of all LD blocks of SNPs tested for ASE also showed significant shifts in the *P* value distributions in both GWA studies (Fig. 5c, d; *P* = 6.80e-07 and *P* = 4.20e-08). Focusing on DCM-specific eQTL, we also observed this shift in the *P* value distributions (*P* < 5.78e-10 in [51] and *P* < 8.99e-12 in [52]). These results indicate that the genomic regions in which we have identified these sQTL, eQTL, and ASE SNPs contain genetic variants regulating splicing and transcription that are important in the development of DCM in the general population. As such these variants could point to biologically relevant candidate genes and polymorphisms in the context of DCM.

Hence, we were interested in whether these genetic variants could be used to improve the prediction of DCM risk. We trained a multilocus genetic risk score (see “Methods”) using DCM GWA data [51], which comprises 909 DCM cases and 2120 controls from Germany. We selected the most predictive variables from covariates (sex and age), eQTL SNPs, sQTL SNPs, and the DCM GWA SNP rs9262636. In a tenfold cross validation we found that the selection based on all candidate variables yielded risk scores with the largest area under the ROC curve (median AUC = 0.70; Additional file 1: Figure S9). A model based only on the GWA SNP yielded a median AUC = 0.65 and a model based on age and sex only resulted in median AUC = 0.63. Overall we observed a moderate improvement of the risk score when including eQTL and sQTL, indicating that these SNP sets encoded relevant information on DCM risk.

### eQTL are enriched for heart GWA SNPs

The sQTL and eQTL detected in this study may also shed light on the underlying biology of other disease/phenotype-associated variants reported in GWAS for the heart. We collected SNPs associated with cardiac phenotypes from the GWA literature [55] and annotated these with our QTL results (Additional file 8) for genes with RPKM >1 in >5% of the samples. Overlap between GWA and eQTL SNPs can be used as functional evidence to prioritize and implicate candidate genes. Overall we have identified eQTL (nominal *P* < 1.0e-05) at 60 of the 298 heart GWA loci, which represents a highly significant enrichment (OR = 3.4, *P* < 2.2e-10, FET) of eQTL for GWA variants. This is many more than identified in the donors only in a previous study [56], probably due to a near doubling in sample size and higher sensitivity of RNA-seq compared to the gene expression microarray platform used previously. In the GTEx data, 39 GWA SNPs were significant eQTL, of which 24 were also identified in our study. When also considering lowly expressed genes, the numbers of GWA SNPs with eQTL in our study increased to 70 (OR = 3.1, *P* = 2.2e-16, FET) and 45 in GTEx. Overall, these results demonstrate the added value of an increased sample size for the interpretation of disease variants. Using a similar strategy, we have also identified ten GWA SNPs that were trQTL (Additional file 9).

Inspecting all loci with eQTL in our study or the GTEx study, we found 19 cases where the GWA SNP was exclusively an eQTL for the candidate gene nominated in the original publication, i.e., the lead SNP of the GWAS was significantly associated with the expression of the candidate gene. In 17 cases, the GWA SNP was an eQTL for the candidate gene as well as an additional gene. These new genes should be considered as possible alternative candidate genes. In more than half of the GWA loci with eQTLs (39) the disease SNP was not an eQTL for the original candidate gene, but for a different gene in the same locus, which should be considered as better candidate genes. One example is the SNP rs2485376, which is associated with the duration of the QTc interval on the electrocardiogram (ECG)—a parameter of cardiac repolarisation [57]. In the original study, *GBF1* was identified as a candidate gene. In our data we did not find evidence of an association between the SNP and *GBF1*, but with *PITX3* (*P* = 4.75E-10). *PITX3* is an interesting candidate, as *PITX2*, a transcription factor from the same family, has been implicated in transcriptional regulation of cardiac ion channel genes [58, 59] and genetic variants close to *PITX2* have been associated with atrial fibrillation [60].

## Discussion

In this work we present the largest heart eQTL data set to date (compare [7, 56]) based on deep RNA-sequencing of DCM patients and non-diseased donors. DCM and non-diseased left ventricular tissue showed marked transcriptome differences. Transcript levels of both protein-coding and long noncoding genes as well as their splicing patterns were altered. This affected many known genes and biological processes involved in DCM (e.g., *TBX20*, *RBM20*) or heart failure (*NPPA*, *NPPB*) and also revealed many novel DCM candidate genes. Differentially expressed genes such as *TBX20* and its target genes constitute a relevant starting point for mechanistic studies to identify the genes whose regulation in failing hearts suggests their biological involvement in disease, which may provide novel leads to study their mechanistic role. Our results provide a rich source of information about the molecular mechanisms that are altered in DCM, including the differential expression of non-coding genes and differential splicing, which previous RNA-seq studies were not able to detect due to a limited number of samples (two DCM cases compared to three non-failing controls [61]). Differences in alternative splicing are particularly interesting as this process has previously been implicated in the etiology of DCM [21, 24, 32]. Our results provide further support for the role of RBM20 in DCM, as we have identified differential splicing of RBM20 targets that we previously identified in rat [24].

The current experimental design has a few limitations with respect to the differential expression analysis due to logistical reasons and challenges related to obtaining human myocardial tissue samples. Ideally the DCM cases and non-diseased controls would have all been recruited in the same centers, sequencing should have been performed in mixed runs, and both populations should have been more deeply phenotyped. We have implemented the following strategies to control the effects of unwanted confounding: the RNA-seq data were generated in the same lab, handled via the same procedures, and analyzed using the same pipeline. The statistical analysis included all known covariates as applicable and we imposed additional thresholds on the fold changes to obtain conservative results. The effectiveness of these considerations was supported by the observation that genes and processes were identified that had previously been associated with DCM. Apart from being a biological validation, this confirmed that the known and unknown sources of technical or biological variation were handled sufficiently. Within the eQTL analyses we addressed any residual confounding by including latent confounder estimates through the PEER procedure [62]. The latter approach has been shown to be a robust and effective method for controlling the influence of latent confounding factors on detection of eQTLs based on multi-center RNA-seq data [6, 63]. Nevertheless, the fact that samples were collected at different centers and sequenced over an extended period in a non-randomized order could still lead to confounding in the comparison of DCM patients and controls, which cannot be ruled out completely.

We showed that there is a widespread effect of genetic variation that affects the regulation of transcription and splicing, which is congruent with the recently published insight that transcription and RNA splicing are the primary links between genetic variation and disease in general [12]. We were able to identify significantly more eQTL in comparison with previously published results from the GTEx project, which is based on post-mortem sections [7]. We showed that the effect sizes and directions were largely concordant between the two studies; however, many eQTL did not reach genome-wide significance levels in the GTEx study, likely due to a slightly smaller sample size or the effects of post-mortem RNA degradation. We also detected genetic variants that affect gene regulation specifically only in DCM patients or in donors, which might be due to an altered *trans* context in diseased and non-diseased tissue. Peters et al. [64] identified cell type- and disease-specific eQTL in immune cells in patients with autoimmune disease, supporting the existence and biological importance of disease-specific eQTL. We detected ten times as many QTL affecting the relative abundance of transcript isoforms (trQTL) in comparison to the GTEx study and showed that this is due to increased sample size, sequencing depth, and the size of the *cis*-window. In agreement with previous studies [6, 7, 42, 43] we showed that QTL variants are frequently located in *cis* regulatory elements, suggesting that these QTL indeed affect promoters or enhancers for the corresponding target gene. In line with previous studies [12] we observed that sQTL are located preferentially close to the exon that they affect. In keeping with results from [65] we observed enrichment of sQTL in different chromatin features such as DNAse I sites and promoters. In addition we also observed enrichment in polycomb-associated regions, which have been shown to affect splicing [66]. Both observations are compatible with the idea that chromatin influences co-transcriptional splicing [67, 68].

We observed allele specific expression for many genes across DCM and non-diseased donor tissues. Differences in allelic imbalance between DCM and donors appear to be small on the individual gene level, yet all differential allelic imbalanced genes combined are enriched for DCM-related processes, as well as differential splicing and miRNA interference. Although we observed ASE in *TTN* regardless of disease status, these sites were not shared between individuals. While truncating variants in *TTN* can lead to nonsense-mediated decay [69], there was no clear pattern emerging from the ASE analysis, probably due to the difficulty of phasing variants across the very large *TTN* transcript. Similar observations were made in a subset of DCM samples [20]. For the DCM phenotype, this could indicate that imbalance shifts towards disease contributing alleles during disease progression. Although allele-specific expression, allelic imbalance, and its potential determinants have been studied genome-wide before [6, 65], to our knowledge this is the first time that this process has been associated with a disease.

By analyzing DCM GWA data we showed that eQTL and sQTL variants are enriched for DCM associations. However, as we focused solely on DCM GWA data it remains open whether this enrichment is specific to DCM or also holds for other diseases and phenotypes. Building on the enrichment results, we showed that QTL variants can be used to derive a multilocus risk score for DCM that outperforms risk scores based on clinical variables and the GWA hit SNP only. Although we apply a ten-fold cross validation, this is not a true replication and is still sensitive to overfitting. In its current form this risk score therefore has limited use for clinical applications. It does, however, demonstrate that QTL variants together encode biological information that significantly improves the prediction of the DCM phenotype. More generally, we found that 20% of all GWA loci for heart-related phenotypes published to date alter gene expression levels. Compared to the 13% identified in the GTEx data set, this increase constitutes a substantial improvement in candidate gene prioritization, an important bottleneck in GWA study follow-ups.

## Conclusions

The RNA-seq-based QTL data set of DCM patients and non-diseased donors generated in this study represents a powerful resource for the whole field of cardiovascular genetics. It revealed marked transcriptome differences between diseased and non-diseased tissue and a widespread effect of genetic variation on the regulation of transcription and splicing. Moreover it allowed for great improvements in GWA candidate gene prioritization, facilitating the elucidation of the mechanisms underlying the genetic basis of common diseases of the heart.

## Methods

### Transcriptome profiling in cardiac samples from donors and patients with DCM

All studies were carried out according to institutional guidelines, and with appropriate informed consent from participants or next of kin. Institutional ethics committees of the centers where the samples were collected reviewed and approved all protocols.

#### Left ventricular samples from patients with DCM

Left ventricular tissue samples from patients with end-stage DCM were retrieved during left ventricular device implantation or/and cardiac transplantation in the period between 1993 and 2011. They were snap-frozen and stored in liquid nitrogen in a tissue bank at the Royal Brompton and Harefield Hospitals NHS Foundation Trust. The set of 128 DCM cases originally considered for this study consisted of end-stage non-ischaemic DCM patients for whom good quality RNA from left ventricular tissue was available for RNA-sequencing analysis. Out of all these included cases, less than 10% report a family history. The diagnosis of non-ischaemic DCM was confirmed from medical records, but additional clinical data were not available. After genotype quality control (see below and Additional file 1: Table S1) 97 samples were used for data analysis.

#### Left ventricular samples from donor hearts

Left ventricular samples were obtained from unrelated organ donors whose hearts were explanted to obtain pulmonary and aortic valves for transplant or valve replacement surgery or explanted for transplantation but not used due to logistical reasons. The 108 samples studied here represent a subset of the 129 samples described previously [56]. The selection was based on the quality of RNA for RNA-seq.

For both cohorts, RNA was extracted from frozen left ventricle with Trizol (Life Technologies) by following the manufacturer’s protocol and subsequently the RNA was quantified using UV spectrophotometry. RNA quality was assessed with the Agilent 2100 Bioanalyser and RNA 6000 reagents. Non-stranded, poly(A)-selected RNA libraries were prepared for sequencing using 4 μg of total RNA as input for the TruSeq RNA Sample Preparation Kit (Illumina).We then generated 2 × 100-bp reads of barcoded cDNA fragments of poly(A) + RNA on a HiSeq 2000 (Illumina) using paired-end chemistry. Six samples were pooled and loaded on three lanes to avoid batch effects and obtain sufficient coverage for splicing analyses.

### Processing of RNA-seq data

The paired-end RNA-seq reads were aligned against the human genome assembly GRCh37 using TopHat version 1.4.1 with option -r 0. This specifies the mate inner distance, which is expected to be zero, since we have 200 bp fragment size and 100-bp reads. In addition we specified option -M that removes multimapping reads before aligning to the transcriptome. The remaining options were set to their default values. We have supplied transcript annotations from Ensembl version 66, which specifies known splice junctions and exon boundaries. In addition we also enabled TopHat’s coverage-based search for novel exons and splice junctions.

### Quantification of the transcriptome

To quantify transcriptome features we have used the Gencode annotation version 19 and augmented it with annotation of the MHRT lncRNA locus [26] and a custom TTN annotation [20]. For gene level quantification we used htseq-count version 0.5.3p3 and the ‘intersection-nonempty’ mode that is suited to quantify overlapping transcripts on different strands. Transcript levels were estimated as fragments per kilobase per million sequenced (FKPM) using cufflinks (version 2.2.1) [70] with the same gene models as above. Exon coverage was determined using intersectBed from bedtools version 2.15.0. The ‘percent spliced in’ index (PSI) [34] was computed using scripts from [35] on non-overlapping exonic parts derived from the Gencode annotation version 19 using the script dexseq_prepare_annotation.py from the DEXSeq R package [2, 9] version 1.8.0.

Heart tissue is mainly composed of cardiomyocytes and fibroblast cells. In order to avoid confounding by cell type heterogeneity in the heart tissue samples we have defined a fibroblast gene expression signature. We analysed RNA-seq data from cultured rat cardiomyocytes and heart fibroblasts in order to identify fibroblast-specific marker genes. Rat cardiomyocytes and fibroblasts were isolated from hearts of neonatal SD rats as previously described with minor modifications [2, 3, 7, 13]. Briefly, 1–2-day-old rats were euthanized and their hearts were excised. Ventricular tissue was minced and incubated in 0.1% trypsin (Sigma) in HBSS (Biochrom) overnight at 4 °C. Five or six digestions for 4 min each were performed with 10 ml of 0.1% collagenase (Worthington) in HBSS. Cells were pooled, collected by centrifugation at 1100 rpm, and resuspended in DMEM supplemented with 10% FCS. To selectively enrich for cardiomyocytes, cells were preplated for 1 h in a T75 flask during which period cardiofibroblasts attached readily to the bottom of the flask. The supernatant was then seeded in 15 cm dishes (~1 × 107 cells/dish) and cardiomyocytes were cultured for 3 days in DMEM supplemented with 10% FCS. Fibroblasts were cultured for two passages in 5 days to increase cell purity by overgrowing non-proliferating myocytes. RNA-sequencing was performed using the same procedures as for the human samples. Reads were processed as the human data using the rat reference genome assembly rn4 and Ensembl version 66. Differentially expressed genes were identified using DESeq [71]. We selected genes with high expression levels in fibroblasts, at least tenfold higher expression in fibroblasts compared to cardiomyocytes, and FDR <0.05. From this list, we selected genes that had human homologs. Using this gene list we computed a fibroblast score for each human sample by summing up the scaled and log10 transformed expression levels. This fibroblast score was subsequently used for adjusting expression levels in eQTL and differential expression analyses.

### Genotyping

#### DCM patients

DNA isolated from peripheral blood samples was used for genotyping on the Affymetrix GW6 platform at the Max Delbrück Center in Berlin according to the manufacturer’s protocol. Genotype calls for 906,600 SNPs were obtained from the Affymetrix genotyping console software version 4.1.4.840 using the birdseed2 algorithm with default settings. Prior to imputation, quality control was performed using GenABEL. We checked for sex mismatches and removed related individuals and individuals with admixed or non-european ancestry (Additional file 1: Table S1). After quality control (QC) we retained 97 DCM patients for the analysis.

#### Non-diseased donors

Genotyping and QC of genotypic data and post-QC processing of the left ventricular samples obtained from donors has been described in detail previously [56]. Genome-wide SNP genotyping was performed using Illumina HumanOmniExpress Beadchips interrogating 733,202 genetic markers. QC was carried out in the GenABEL package in the statistical programming language R using default settings. Only the data of the 108 samples for which RNA-seq data were generated were used in the present study, all of which passed QC and were part of the original 129 samples used in the original study [56].

Using data of both populations we checked for population structure and computed the first three principal components for inclusion in our models as covariates.

As independent quality control for both DCM patients and donors, we obtained SNP calls from RNA-seq data for known SNP positions in exons based on the 1000 Genomes data set phase 1 version 3. For this analysis we ran TopHat with very stringent read mapping criteria to avoid artifacts from misaligned reads. We have then selected all SNP positions for which we were able to obtain high confidence genotype calls (per sample, for each genotype call, median PHRED score >30, covered by at least 30 reads, and reference and alternative allele freq in {0; 0.5; 1} ± 0.25). We used genotypes at 2043 positions for which data were available from both platforms and RNA-seq to compare the accuracy (fraction of correct genotype calls) of the two platforms (Additional file 1: Table S7). To rule out potential effects of allele specific expression, we computed accuracy also for individuals with heterozygous array genotypes only. In addition, we computed the non-reference accuracy (fraction of correct minor allele genotype calls).

### Genotype imputation

Since the genotypic data in DCM patients and donors was obtained on different genotyping arrays, which have an overlap of around only 200,000 SNPs, we used genotype imputation to increase the resolution of our genetic map. The cases were typed on the Affymetrix GW6 array with 906,600 SNPs, while controls were typed on the Illumina HumanOmniExpress with 733,202 SNPs. Both genotype data sets were first filtered according to the following quality criteria. In each data set, we required SNPs with minor allele frequency (MAF) greater than 5% to have a call rate of at least 95% and SNPs with lower MAF to have a call rate of 99%. Moreover the test for Hardy–Weinberg equilibrium had to be *P* > 0.0001. We selected the set of 195,386 SNPs that were typed on both platforms and passed the quality criteria as the input for the genotype imputation.

The reference haplotypes were obtained from the 1000 Genomes data set phase 1 version 3 that comprises reference haplotypes for 1092 individuals. We applied shapeit v1 for the prephasing of the genotypes. We used impute v2 for the actual genotype imputation.

We assessed the quality of the imputed genotype calls using two data sets. The first data set was based on the genotype calls from the RNA-seq described in the section genotyping. The second data set consisted of all genotypes from the SNP array that were not used for the imputation because they were specific to one of the two arrays. Imputation quality was measured as overall genotype accuracy (fraction of correct genotype calls), non-reference accuracy (fraction of correct minor allele genotype calls), and imputation efficacy (fraction of individuals with genotype confidence *P* > 0.95). Additional file 1: Figure S3 shows the imputation quality based on the RNA-seq data. Overall, we achieved a good efficacy (Additional file 1: Figure S3a) and also a good accuracy (Additional file 1: Figure S3b). The non-reference accuracy, however, shows a bimodal distribution with very high values, but very low values in some instances. This is expected to occur for low MAF variants when all individuals are assigned the major allele. Indeed Additional file 1: Figure S3d shows that the very low non-reference accuracy values occur at low MAF. Therefore, to achieve good non-reference accuracy, we use imputed variants with MAF >0.1 only. Similar results were obtained using the SNP array-based evaluation (data not shown).

In order to avoid artifacts in QTL analyses caused by rare genotypes that coincide with outliers in the expression data, we substituted homozygous minor alleles that occurred less than three times by the heterozygous genotype.

### Differential expression

Gene expression counts were normalized using a quantile-based scaling method [72]. Differential gene expression was determined from the normalized gene expression count matrix as follows. The normalized counts were log transformed and adjusted for the clinical covariates using a linear model. For each gene we computed residuals from the linear model and added the mean expression level to preserve the information about the absolute expression values. Differential expression between DCM cases and donors was assessed using the Wilcoxon–Mann–Whitney test. In addition we required large expression differences (absolute value of the log fold change greater than log(1.2)) to avoid spurious findings.

### Differential exon usage

PSI values were calculated for 245,309 counting bins. Only those counting bins with 0 < PSI < 1 for all samples were considered for the analysis; i.e., counting bins that are excluded or included in all samples are not of interest. We tested for differential exon usage between the DCM cases and the donors using the two nested linear models:

$$ \mathrm{Full}:\mathrm{PSI}\sim \mathrm{DCM}+\mathrm{fibroblast}\  \mathrm{score}+\mathrm{age}+\mathrm{RIN}\ \mathrm{score}+\mathrm{sex} $$

$$ \mathrm{Reduced}:\mathrm{PSI}\sim \mathrm{fibroblast}\  \mathrm{score}+\mathrm{age}+\mathrm{RIN}\ \mathrm{score}+\mathrm{sex} $$

and the likelihood ratio test statistic. To focus on biologically relevant hits, we used a conservative cutoff for the estimated effect size of 0.1 corresponding to a 10% difference of PSI values.

Similar approaches based on linear regression of PSI values have been used for the analysis of sQTL [41]. To assess the expected sensitivity and false discovery rate of this approach, we performed a simulation study. For each sample we obtained the total read counts for each exon. We removed all exons that had zero counts in more than 10% of the samples. We fitted a negative binomial distribution to these counts excluding counts larger than the 90th percentile. Then we simulated total read counts for all exons from this distribution. In the next step we simulated the actual inclusion rates (PSI) for all exons from a uniform distribution. Then we selected 10% of all exons to be differentially used and modified the actual PSI values by 10% in the samples of the case group. Finally, we drew the number of inclusion reads per exon in that sample from a binomial distribution with the total reads of the exon as size parameter and the actual PSI of the sample as success probability and computed the simulated PSI values as inclusion reads over total reads. We applied the linear regression model (without the covariates) to the simulated PSI values and predicted the differential exons using the criteria defined above. The predictions were compared to the simulated differential exons to compute the sensitivity (TP/(FN + TP)) and false discovery rate (FP/(FP + TP)). The simulation was repeated 100 times. Additional file 1: Figure S10 shows that the false discovery rate is very low (<1%) and the sensitivity is about 22%, indicating a conservative behavior of the method.

### eQTL mapping

We used all 205 samples from DCM patients and donors for the eQTL analysis. To associate gene expression values with genotypes we applied the same procedure that was used in the GTEx study [7]. Briefly, we transformed read counts to RPKM values (reads per kilobase of transcript per million mapped reads) and selected all genes that had RPKM >0 in at least 50% of all samples. We applied quantile normalization across all genes to obtain comparable gene expression distributions between samples. Subsequently we quantile normalized the expression values of each gene across samples to a standard normal distribution to minimise the effect of outliers. Ties in the ranking were resolved randomly. We used the PEER method [62] to correct for hidden confounding factors in the expression data. Similar to [6] we evaluated the impact of using different combinations of covariates on the detection rate of *cis* eQTL. We compared the number of genes with *cis*-eQTL (*P* < 1e-6) using no covariates, measured covariates (sex, age, fibroblast score, RIN score, center), the first three principal components of the genotype data, measured and genetic covariates, as well as 5 to 25 PEER factors (Additional file 1: Figure S11). Note that the DCM status is implicitly adjusted for by the center variable, as all DCM cases were recruited in London. For the final eQTL analysis we used 25 PEER factors and the measured covariates listed above. eQTL were identified using MatrixEQTL [73] to test all *cis* SNPs within a distance of 1 Mb of a gene. In Table 2 and in the comparison with the GTEx study we used nominal *P* < 1e-5 as significance threshold, which corresponds to a Benjamini–Hochberg adjusted FDR of 0.1%.

### DCM/donor-specific eQTL

DCM- or donor-specific eQTL were identified using a two-step approach. First we performed separate eQTL analyses in DCM patients and in donors using the same method as described above. To avoid imputation artifacts we only considered genotypes that were measured on both platforms. In addition we focused this analysis on transcripts with Refseq models only. We selected all SNP–gene pairs for which an eQTL was detected (FDR <0.05) only in one of the two analyses as candidate-specific eQTL. In a second step we performed an analysis using nested linear models to rule out that the eQTL was not detected due to power issues. We compared a full model that includes separate slopes and intercepts for each group, with a reduced model that only contains one common slope for both groups and intercepts for both groups. Log transformed normalized gene expression data adjusted for all available covariates y_ij_ of gene i in sample j was modeled as a linear function of genotype dosage x_kj_ of SNP k and a group indicator variable g_j_. Specifically the two models were:

$$ \mathrm{Full}:{\mathrm{y}}_{\mathrm{ij}}={\mathrm{g}}_{\mathrm{j}}\left({\upbeta}_0+{\upbeta}_1{\mathrm{x}}_{\mathrm{ij}}\right)+\left(1\hbox{-} {\mathrm{g}}_{\mathrm{j}}\right)\left({\upbeta}_2+{\upbeta}_3{\mathrm{x}}_{\mathrm{ij}}\right)+{\upvarepsilon}_{\mathrm{ij}} $$

$$ \mathrm{Reduced}:{\mathrm{y}}_{\mathrm{ij}}={\mathrm{g}}_{\mathrm{j}}\ {\upbeta}_0+{\upbeta}_1{\mathrm{x}}_{\mathrm{ij}}+\left(1\hbox{-} {\mathrm{g}}_{\mathrm{j}}\right){\upbeta}_2+{\upvarepsilon}_{\mathrm{ij}} $$

with ε_ij_ being the iid normal error term. Models were compared using the likelihood ratio test as in regular ANOVA. DCM- or donor-specific eQTL were identified using FDR <0.05. In addition we required that zero was in the 95% confidence interval for the estimates of β_1_ or β_3_ to make sure that only one of the two slopes is significantly different from zero.

We applied the Armitage trend test (implemented in GenABEL [74]) for differences in allele frequencies to assess if DCM- or donor-specific eQTL were due to systematic differences in allele frequencies between the two groups.

### sQTL mapping

For the sQTL analysis we used exon expression levels of exon counting bins defined as in [33] using all transcripts from GENCODE v19 that also had a Refseq model.

We used an adaptation of the DEXSeq model [33] for sQTL analysis. The original DEXSeq model was designed for rather small data sets, so it was too slow for analyzing large numbers of SNPs for each exon and too sensitive for large sample sizes, detecting very small effects that are likely false positives. To reduce runtime, we used a regular linear model with Gaussian error terms instead of a generalized linear model for count data. We therefore quantile-normalized exon expression levels across all exons within each sample such that they follow a standard normal distribution. We encoded SNP genotypes as factors by rounding the imputed genotype dosages. The normalized exon expression levels y_ijl_ of exon l in gene i and sample j is modeled by two nested models as:

$$ \mathrm{Full}:{\mathrm{y}}_{\mathrm{ij}\mathrm{l}}={\upbeta}_0+{\upbeta^{\mathrm{G}}}_{\mathrm{ij}}+{\upbeta^{\mathrm{E}}}_{\mathrm{il}}+{\upbeta^{\mathrm{S}}}_{\mathrm{ik}}+{\upbeta^{\mathrm{E}\mathrm{S}}}_{\mathrm{il}\mathrm{k}}\ {\updelta}_{\mathrm{ll}'}+{\upvarepsilon}_{\mathrm{ij}} $$

$$ \mathrm{Reduced}:{\mathrm{y}}_{\mathrm{ij}\mathrm{l}}={\upbeta}_0+{\upbeta^{\mathrm{G}}}_{\mathrm{ij}}+{\upbeta^{\mathrm{E}}}_{\mathrm{il}}+{\upbeta^{\mathrm{S}}}_{\mathrm{ik}}+{\upvarepsilon}_{\mathrm{ij}} $$

where β_0_ is an intercept, β^G^_i_ can be thought of gene expression level in sample j, β^E^_il_ can be thought of as the average difference of exon l to the gene expression level, β^S^_ik_ represents the contribution of the genotype of SNP k, β^ES^_ilk_ δ_ll’_ is an interaction term representing the difference of the exon expression levels between genotypes at SNP k for exon l′ and eps_ijl_ is the iid error term, following a normal distribution. For each exon l′ and each SNP k in a window of ±1 Mb of the gene, we computed the likelihood ratio test for the comparison of the full model and the reduced model. The full model is including the interaction term β^ES^_ilk_ that is multiplied by an indicator variable δ_ll’_ , which is one if l = l′ and zero otherwise. To evaluate the statistical significance of the likelihood ratio statistic we used the F-distribution with the appropriate degrees of freedom depending on the number of exons per gene and the number of observed genotypes. We tested only counting bins that were located within exons that were annotated as alternatively spliced by Ensembl.

To rule out spurious *cis* sQTL associations that might arise if the genotypes of sQTL SNPs were correlated with expression of the DCM-related *trans*-acting splicing factor *RBM20*, we checked the correlation between *RBM20* expression and all *cis* sQTL SNPs (potential *trans* eQTL for *RBM20*). We report the 1 – **π**_**0**_ estimate [37] as the upper limit of the percentage of SNPs that might be false *cis* sQTL because of confounding by *RBM20* expression.

### Transcript ratio QTL mapping

We used isoform quantifications based on cufflinks for the transcript ratio QTL mapping. As previously described, we selected only transcripts with FPKM >0.01 and only genes with at least two transcript isoforms expressed [7]. To make the results comparable with the exon-based sQTL analysis we tested all SNPs in the range of 500 kb from the gene for association. We used sQTLeeker to test association between SNPs and transcript ratios [41]. This method is based on multivariate analysis of variance and tests how well the genotype classes can explain the variation of the samples on the simplex defined by the relative transcript isoform expression levels. Significance was determined by permutations and subsequent control of the false discovery rate (FDR <0.05).

Moreover, we analyzed the effect of the total read coverage, sample size, size of the *cis* window, and covariate adjustment on the detection rate of trQTL. Our main goal was to obtain numbers that are comparable to the GTEx left ventricle data set, which comprises 83 samples assuming a read coverage of 80 million, which is close to the median of 82.1 million of all GTEx samples [7]. Here we used numbers from GTEx analysis version 4, as these results are only reported in the paper and not on the website. To reduce the computational burden of transcript isoform quantification and sQTL seeker analysis we restricted ourselves to chromosome 20. We sampled 1.7 million reads from chromosome 20, assuming that the number of aligned reads is distributed among chromosomes according to their lengths (chromosome 20, 63,025,520; chromsomes 1–22 and X, 3,036,303,846). The 83 samples were randomly selected from the group of donors to remove possible influence of the disease state. In addition we restricted the radius for *cis* SNPs to a maximum of 5 kb. We also adjusted transcript isoform expression levels for the measured covariates fibroblast score, age, RIN score, sex, and contributing clinical center. We first computed mean transcript expression levels and then added an offset of 1 and log transformed the isoform expression levels. Using these values we performed linear regression against the covariates and obtained the residuals from the model. Finally we reversed the log transformation, subtracted the offset, and added the means to obtain adjusted isoform expression levels. All negative values were set to zero.

### SNP level functional analysis of QTL

To determine whether QTL SNPs preferentially occur in certain functional elements, we have annotated all SNPs that were tested for eQTL and sQTL with features based on gene models from GENCODE v19 and *cis* regulatory elements that were determined based on DNA sequence and chromatin state annotations [45] for the left ventricle of the heart obtained from the Roadmap Epigenomics project [44]. To do so, we built a simple logistic regression model:

$$ \log \left(\frac{P_i}{1-{P}_i}\right)={\beta}_0+\sum_{j\in P}{\beta}_j{x}_{ij} $$

to predict which SNP is ‘causal’ for a target from its functional annotation. We considered each pair of SNP and target, i.e., exon for sQTL and gene for eQTL analysis, as a data point. Each data point i is a tuple (y_i_, x_i_), where x is a binary vector (x_i1_,.., x_ip_) indicating whether the SNP–target pair is annotated with feature j, and y_i_ indicating whether the SNP is the most significantly associated SNP for the target. This simple model assumes that the best SNP is also the ‘causal’ SNP for each target. Since other significantly associated SNPs might also be causal and thus functionally relevant, or the causal SNP might not be the most significantly associated SNP, these mislabeled data points might dilute the enrichment results when annotated as ‘not causal’. Therefore, we removed all data points where the SNP was also significantly associated with the target but not the top hit. The distance between SNP and target is an important predictor for QTL [42], so we grouped the distances into five bins of size 10 kb, starting from 1 bp, and used these features for sQTL and eQTL analysis. For both QTL analyses we used chromatin state annotations from the 25 state chromHMM segmentation from roadmap. In particular, we used the states 1) heterochromatin; 2) TSS; 3) bivalent promoter; 4) promoter; 5) DNAse; 6) polycomb; 7) weakly transcribed. Additionally, for eQTL analysis, we annotated whether the SNP was in the 8) promoter region of the target gene; 9) in an exon of the target gene; or 10) in an intron of the target gene. And specifically for sQTL analysis, we annotated 11) whether the SNP was located directly within the target exon; 12) whether the SNP was located in the neighboring intron upstream or 13) downstream of the target exon; 14) whether it was located in the neighboring intron upstream or 15) downstream; 16) exonic splice enhancers (ESE) by matching hexamer sequences defined by [46] to transcript sequences. We estimated the model parameters as well as their standard errors, and tested each of the hypotheses β_j_ = 0, while controlling for the other variables, using the likelihood ratio test as implemented in the glm function of R using the binomial family and logistic link function [75].

### Association of QTL with DCM

To assess the association between regulatory variants and DCM disease risk we analyzed the results of two genome-wide association studies for DCM from a German [51] and a French [52] population. Enrichment for DCM GWAs was assessed using an approach that was initially developed to analyse gene sets [54]. We tested whether the distribution of GWA *P* values for QTL variants is different from the distribution for tested variants without QTL. Since these studies and our own study were carried out on different genotyping platforms, we considered blocks of high linkage disequilibrium (LD) in the CEU reference population that were tagged by SNPs in our study and the DCM GWA as basic units of analysis. LD blocks were defined using SNAP [53] with Rsq >0.6. Each LD block was classified as QTL when it contained at least one SNP with a QTL, and the smallest DCM GWA *P* value of all SNPs within the LD block was considered. The significance of the difference of *P* value distributions was assessed using a one-sided Wilcoxon–Mann–Whitney test with the alternative hypothesis that *P* values of LD blocks with QTL are smaller. To assess whether our approach was sensitive towards outliers that would be selected in the approach considering the minimal *P* values, we also repeated the analysis choosing the second smallest *P* value for each LD block, leading to similar results (data not shown).

### DCM risk score

We estimated and evaluated different multilocus genetic risk models trained from varying sets of input variables. The original DCM GWA [51] genotype data of 292,367 SNPs for 909 DCM cases and 2120 control was generously provided by the authors. Our method is based on feature selection by regularized logistic regression (LASSO) as implemented in the glmnet R package [76]. We prepared sets of candidate variables for selection into the risk model. The following SNP sets were considered: 11,771 SNPs with eQTL (*P* < 10e-5), 9134 SNPs with sQTL (FDR <0.05), one GWA SNP (rs9262636), and the empty set. Furthermore we considered combinations of eQTL + GWA, sQTL + GWA, eQTL + sQTL + GWA. All sets were extended to include the covariates sex and age. To evaluate the risk model we performed tenfold cross-validation. We used the area under the receiver operator characteristics (ROC) curve as performance measure. In each fold we reserved 10% of the data for testing and used the remaining 90% for training of the model. The lambda parameter, which indicates the weight of the L1 penalty in the logistic regression model, was determined in a second nested cross-validation on the training data. We used the largest lambda, which was within one standard error of the maximal training AUC as recommended [77] to obtain parsimonious models.

### Analysis of heart GWA SNPs

To assess the value of our eQTL and sQTL results for nomination of candidate genes for mediating the effect at loci identified in GWA of heart-related traits, we obtained published GWA results from the GWAS catalog (accessed 11.12.2015). We selected all traits that were annotated as heart disease (EFO_0003777) or cardiovascular measurement (EFO_0004298) in the experimental factor ontology. We removed cardiovascular measurements that were not directly related with the heart. For the eQTL we intersected the GWA loci with eQTL data of both our own study and the GTEx study. For each of the studies, we used proxies (LD >0.8) for GWA SNPs that were not in the dataset. To avoid double counting because of LD, we selected only the best proxy SNPs for each pair of GWA SNP and potential *cis* eQTL gene. LD information was obtained from the SNAP database [53]. sQTL were analyzed in the same way, but using only sQTL data from our study. For eQTL-GWA analysis we used genes with RPKM >1 in >5% of the samples, to select candidates that are amenable to biological follow-up analysis. Enrichment of heart GWA SNPs among eQTL was assessed as follows. We considered all SNPs tested for *cis* eQTL for the selected genes as the basic population. Then we classified each SNP as eQTL (*P* < 1e-5) or non-eQTL and GWA (GWA SNP or its best) or non-GWA. Finally, we applied Fisher’s exact test to determine the significance.

### Allele-specific expression analysis

To characterize the allele-specific expression (ASE) in each individual, we performed an allelic imbalance analysis analogous to previously published work [6, 78] and outlined in detail in a best practices article [79]. Briefly, the analysis was based on binomial testing of each allelic ratio of heterozygous sites (as determined from the Illumina microarray genotyping data) within each individual. Sites prone to allelic mapping bias were excluded: 1) sites in regions with low mappability according to the mappability track of UCSC (50 bp mappability <1 implies that the flanking region of the site is non-unique in the genome); 2) sites for which simulated overlapping 50-bp reads show >5% difference in the mapping of reads that carry the reference or non-reference allele (simulation results kindly provided by the GEUVADIS consortium [6, 78]). We adhered to strict quality settings in calling genotypes from the raw RNA-seq reads, requiring a PHRED base quality score larger than 30 and a coverage of at least 30 reads for each site. Additionally, only sites where both alleles are observed in the RNA-sequencing data were considered to ensure that the observed genotype for the site is truly heterozygous. To correct for any remaining genome-wide mapping bias in addition to GC bias, average reference allele ratios were calculated for each individual. Using these expected ratios, a binomial test of the reference and non-reference allele counts was performed. To account for large differences in expression, reflected in large differences in total allele counts, driving the results, all sites were resampled to the mean total allele count of all heterozygous sites. *P* values were subsequently corrected for multiple testing using the q-value method [37] from the qvalue package in R.

To summarize results afterwards, for all sites (that are heterozygous in at least one individual) we calculated how many individuals are heterozygous and how many show allelic imbalance, both in the total set (*n* = 205) as well as in the donors (*n* = 108) and DCM (*n* = 97) samples separately. Allelic imbalance differences between DCM and donors were calculated in two ways. Difference in number of imbalanced individuals was calculated using a Fisher’s exact test (FET; ‘FET *P* value’ abbreviated as ‘FET P’). The difference in imbalanced allele was determined using a test of proportions. In assessing differentially imbalanced sites between DCM cases and non-diseased donors we applied LD pruning on the set of imbalanced variants using results from SNAP (1000 Genomes; R2 > 0.8) to keep only independent variants.

Functional enrichment of imbalanced sites was performed using the NEXUS variant annotation tool [80]. Additionally, overlap with known truncating variants and known imprinted genes (source http://www.geneimprint.com/site/genes-by-species.Homo+sapiens; accessed 14-01-2016) was determined. To assess co-occurrence of miRNA binding sites, only conserved predicted sites from TargetScan 7.0 were used (accessed 30-03-2016). Overrepresentation of eQTL and differential splicing identified in the present study was determined using odds ratios with confidence intervals. Functional enrichment of associated genes was performed using the topGO package in R, using the parent-child algorithm [81] with a minimum node size of 5.

## Additional files

Additional file 1: Supplemental figures and tables. (PDF 1734 kb)

Additional file 2: Differential gene expression of protein-coding genes between DCM patients and controls. The table contains results from a Wilcoxon–Mann–Whitney test (column W) for differential expression based on adjusted RPKM values. We report *P* values (column P) and Benjamini–Hochberg adjusted *P* values. The columns *Control* and *DCM* indicate the means of the RPKM values in each condition. The column *Differential* indicates whether the adjusted *P* value was less than 0.05 and the fold change was less than 0.8 or greater than 1.2. The column DCM candidate indicates whether the gene was a DCM candidate based on [20] and listed in Additional file 1: Table S6. The column *TBX20 target* indicates whether the gene has a TBX20 binding site [31] transferred from mouse to human with coordinate liftover within 10 kb around the gene and *miR-22 target* indicates whether a gene is a predicted conserved target of miR-22 according to targetscan. (XLSX 1427 kb)

Additional file 3: Differential gene expression of noncoding genes between DCM patients and controls. The table shows results from a Wilcoxon–Mann–Whitney test (column W) for differential expression based on adjusted RPKM values. We report *P* values (column P) and Benjamini–Hochberg adjusted *P* values. The columns *Control* and *DCM* indicate the means of the RPKM values in each condition. The column *Differential* indicates whether the adjusted *P* value was less than 0.05 and the fold change was less than 0.8 or greater than 1.2. (XLSX 693 kb)

Additional file 4: Differential exon usage between DCM patients and controls. Exon usage was quantified by the percent spliced in index (PSI) and tested for differences using a linear model. The table reports the positions (chromosome, start, end) of the tested exonic parts, the gene ids, the test statistic (t-statistic), the estimated effect beta, which can be interpreted as the difference of mean PSI values of cases and controls, and the corresponding *P* values and FDR. We define differentially used exons when |beta| >0.1 and FDR <0.05. The column DCM candidate indicates whether the gene was a DCM candidate based on Additional file 1: Table S6. The column *has rbm20 binding* indicates whether a RBM20 Clip-seq site was found at the exonic part. (XLSX 18691 kb)

Additional file 5: DCM and donor specific eQTLs. The table lists significant DCM and donor-specific eQTLs, including information on the gene symbol, identifier of the SNP, group-specific (DCM or control) estimates of the genotype effect, along with the higher and lower bound of the 95% confidence interval and *P* values for the estimated effect being different from zero. In addition, the F statistic, *P* value and Benjamini–Hochberg adjusted *P* value of the nested linear model are reported. The column *Specific* indicates whether the eQTL was specific for DCM (cases.only) or donors (controls.only) according to the definition in the “Methods” section. (XLSX 103 kb)

Additional file 6: Sites with allele-specific expression. The table contains information on SNPs with allele-specific expression, including SNP identifier, position, reference and alternative allele, number of heterozygous individuals, number of individuals with significant allele specific expression, average expressed allele frequencies over individuals, gene identifiers, and gene symbols. The column DCM candidate indicates whether the gene was a DCM candidate based on Additional file 1: Table S6. (XLSX 592 kb)

Additional file 7: Differences in relative numbers of imbalanced individuals between DCM cases and donors. The table contains information on SNPs with differences in allele-specific expression between DCM patients and donors, including SNP identifier, position, reference and alternative allele, number of heterozygous individuals, individuals with ASE, percentage of individuals with allele-specific expression globally and separately for DCM patients and donors. In addition, the table shows *P* values for the binomial test for a difference of the percentage of individuals with allele-specific expression between DCM patients and donor samples. The gene in which the SNP is located is identified by Ensembl gene id and gene symbol. The column DCM candidate indicates whether the gene was a DCM candidate based on Additional file 1: Table S6. (XLSX 29 kb)

Additional file 8: Annotation of heart-related GWA SNPs with eQTLs. The table contains heart GWA loci annotated with eQTL information. The columns are: Proxy SNP identifier, GWA SNP identifier, GWA trait, Pubmed ID reporting the GWA study, GWA *P* value, gene symbols of the reported candidate genes, Distance between proxy and GWA SNP, RSquared and DPrime between proxy and GWA SNP, eQTL gene identifier, eQTL gene symbol, eQTL gene type, Genotype effect estimate (beta), eQTL *P* value, genotype effect estimate in GTEx (beta GTEx), eQTL *P* value in GTEx, gene identifiers of the reported candidate genes, percentage of samples with RPKM >1, indicator of significant expression (percentage of samples with RPKM >1 at least 5%). (XLSX 89 kb)

Additional file 9: Annotation of heart-related GWA SNPs with trQTLs. The table contains heart GWA loci annotated with trQTL information. The columns are: Proxy SNP identifier, GWA SNP identifier, GWA trait, Pubmed ID reporting the GWA study, GWA *P* value, gene symbols of the reported candidate genes, Distance between proxy and GWA SNP, RSquared and DPrime between proxy and GWA SNP, trQTL gene identifier, sQTLseeker test statistic (F), trQTL *P* value, the identifiers of the two most abundant isoforms of the trQTL gene, gene symbol of the trQTL gene, and identifiers of the reported candidate genes from the literature. (XLSX 62 kb)
