# Supplementary material for: Natural genetic variation of the cardiac transcriptome in non-diseased donors and patients with dilated cardiomyopathy
Source: Genome Biol. 2017 Sep 14;18:170. doi: 10.1186/s13059-017-1286-z (PMC5598015; doi:10.1186/s13059-017-1286-z)
Supplement: Supplementary file 1 — Supplemental figures and tables. (PDF 1734 kb) [file 13059_2017_1286_MOESM1_ESM.pdf]

SUPPLEMENTAL MATERIAL

Supplemental Notes

Comparison to the GTEx study

Here we provide a summary of the comparisons to the GTEx study in a compact way.

eQTL detected from combined population
We systematically compared our results to eQTL from left-ventricular tissue of the GTEx project [7], which comprises 190 samples in version 6. For the comparison on the SNP level, we selected the most significant marker for each gene with cis eQTL in our study (nominal P < 1e-5). Among the top SNPs, 82% were also analyzed in the GTEx study. Of these, 97% had concordant allelic effects (Supplemental Figure 3) although only 40% reached the significance threshold in GTEx. The larger number of eQTL detected in our study is most likely due to reduced statistical power caused by a slightly smaller sample size or the use of post-mortem tissues in the GTEx project. Using Storey's q-value method [34], we estimated that 93% of eQTL are actually shared. Conversely, we have analyzed 18% of the top GTEx SNPs for genes with cis eQTL, of which 72% were significant in our study, 97% had concordant allelic effects and 92% were estimated to be shared.

eQTL detected from individual populations
Given the presence of DCM specific eQTLs we also performed comparisons between the GTEx results and eQTL results obtained using the non-diseased donor samples and the DCM patients separately. The concordance of the direction of the genotype effect was between 96 – 98% in all comparisons (Supplemental Figures 5-6, Supplemental Table 8). The fraction of shared significant eQTLs detected in the donor samples was slightly higher (60%) compared to the DCM samples (55%). Using the GTEx eQTLs as basis for the comparison, we found 43% shared eQTLs in the donor samples and 47% shared eQTLs in the DCM samples. These numbers are lower than in the comparison of GTEx eQTLs to the results of the full study, where 72% were shared, most likely due to the reduced sample size in the separate analyses.

trQTL
In our study, we have identified 2,874 trQTL (14.6% of tested gene). In comparison to the GTEx study (242 trQTL, ~1.2% of tested genes), we identified over 10 times as many trQTL. To investigate the possible factors leading to this increase in detection rate, we matched our data set to the GTEx data set in terms of read depth, sample size and size of the *cis* window (see Methods). In this matched analysis we estimated that 1.5 % of genes have trQTL (Table 2), which is in accordance with GTEx.

Annotation of GWA hits

Overall we have identified eQTL (nominal P < 1.0e-05) at 60 of the 298 heart GWA loci, which represents a highly significant enrichment (OR = 3.4, P < 2.2e-10, FET) of eQTL for GWA variants. In the GTEx data, 39 GWA SNPs were significant eQTL of which 24 were also identified in our study. When also considering lowly expressed genes, the numbers of GWA SNPs with eQTL in our study increased to 70 (OR = 3.1, P= 2.2e-16, FET) and 45 in GTEx.

Supplementary Figures

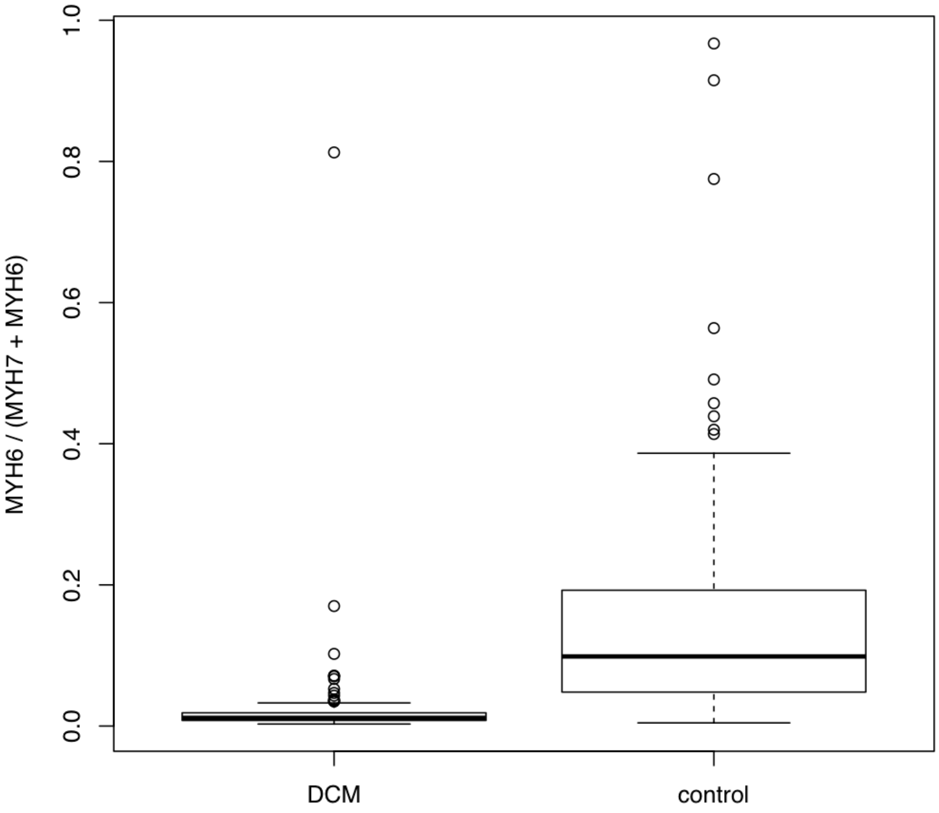

Supplemental Figure 1: *Differential expression between DCM patients and controls at the myosin heavy chain locus*. Boxplots show the abundance of MYH6 transcripts relative to all MYH6 and MYH7 transcripts in DCM patients and healthy controls.

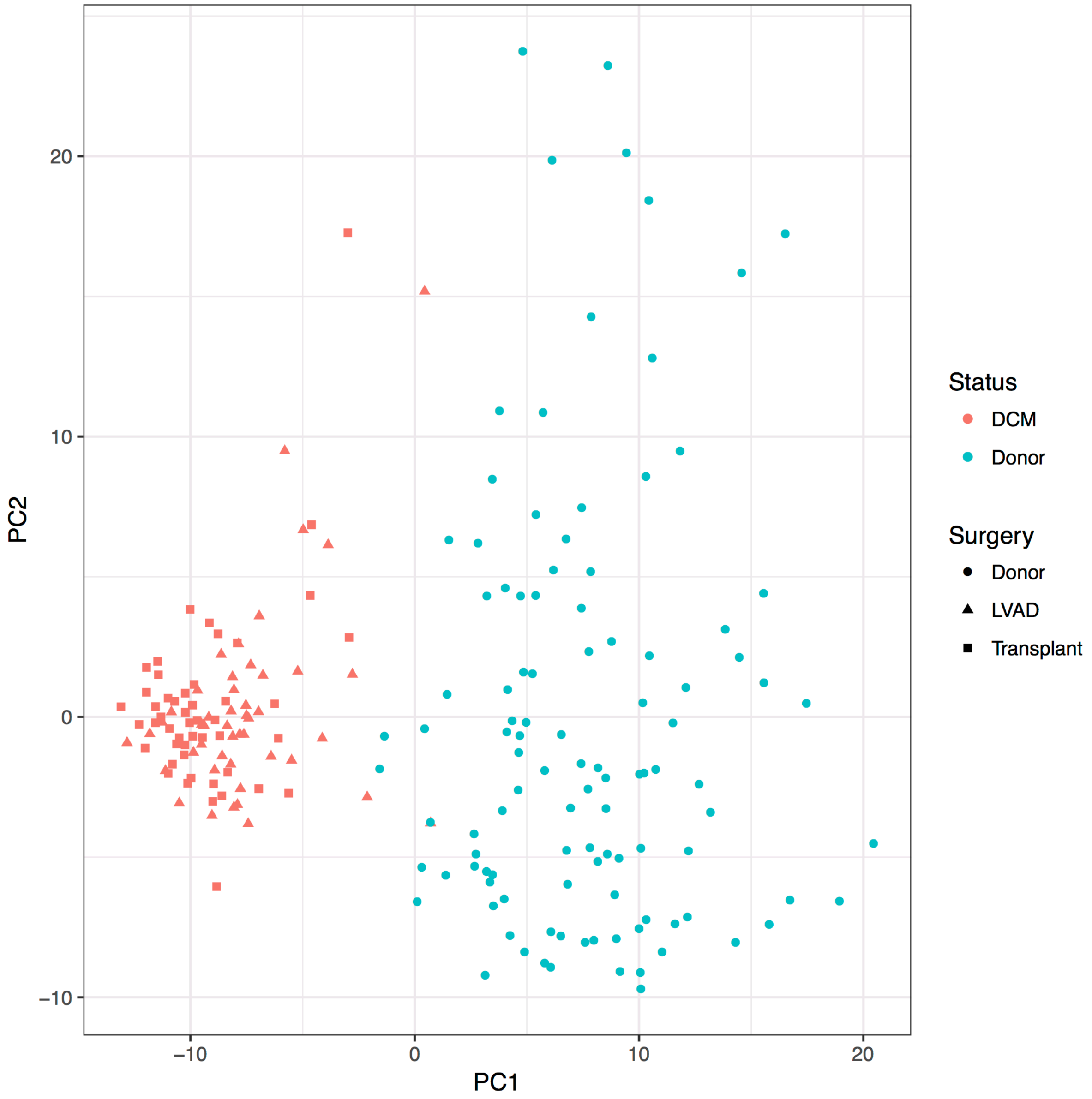

Supplemental Figure 2: *Principal components analysis of DCM patients and donor samples*. The scatter plot shows the projection of the expression data on the first (x-axis) and second (y-axis) principal components. Color encoding shows the status (DCM or Donor) and the shape of the symbol indicates the type of surgery that was performed on the DCM patients.

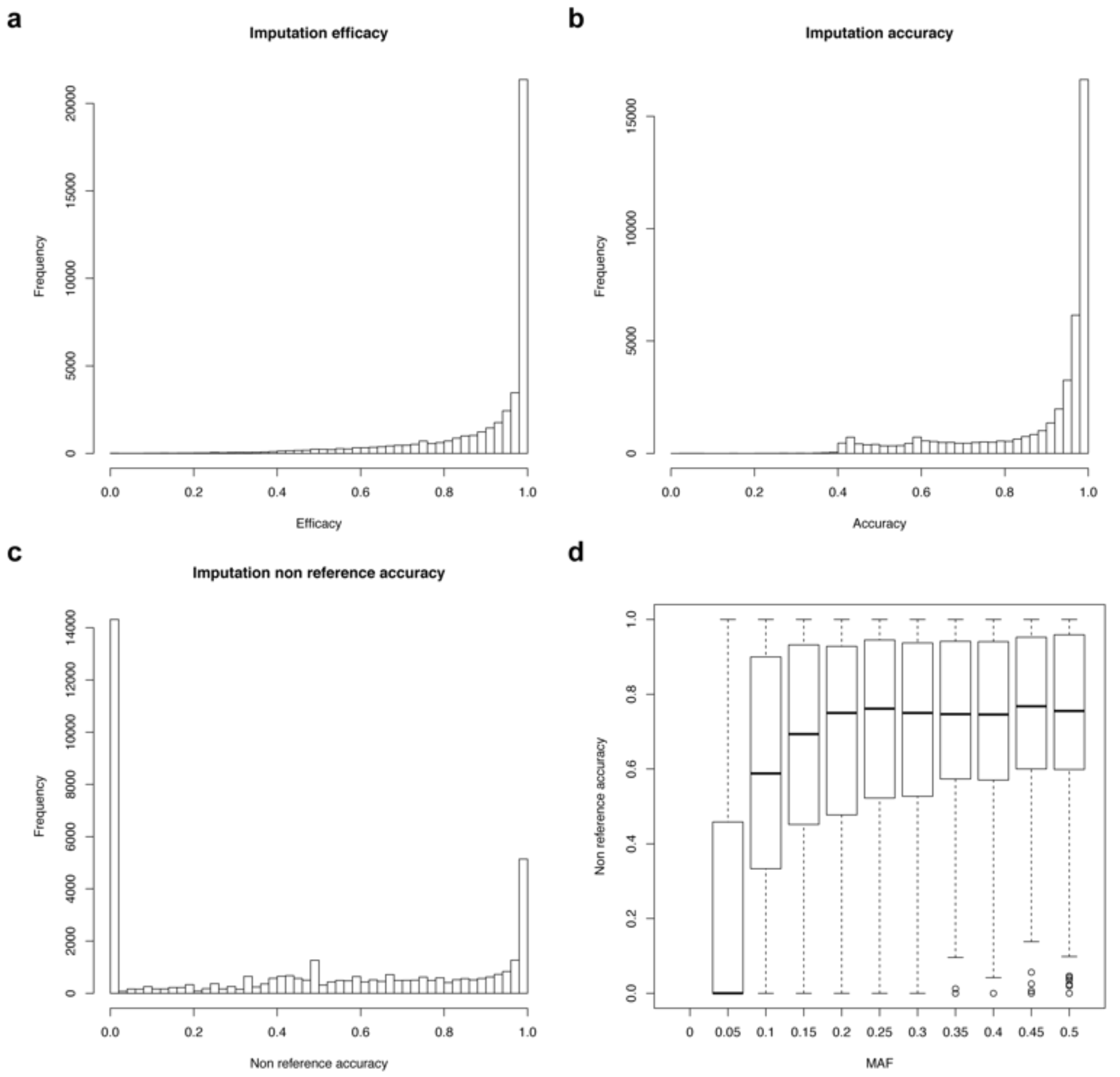

Supplemental Figure 3: *Assessment of genotype imputation quality.* We have used RNA-seq reads at common variant positions in exons with high coverage and not in allelic imbalance to derive a high confidence set for the evaluation of imputation quality. We measured **a)** imputation efficacy as the fraction of confidently imputed samples per SNP, **b)** accuracy as the fraction of correct genotype calls per SNP, **c)** non reference accuracy as the fraction of correct non reference genotype calls per SNP. The boxplot in **d)** shows the non reference accuracy for different minor allele frequency (MAF) bins of width 0.05.

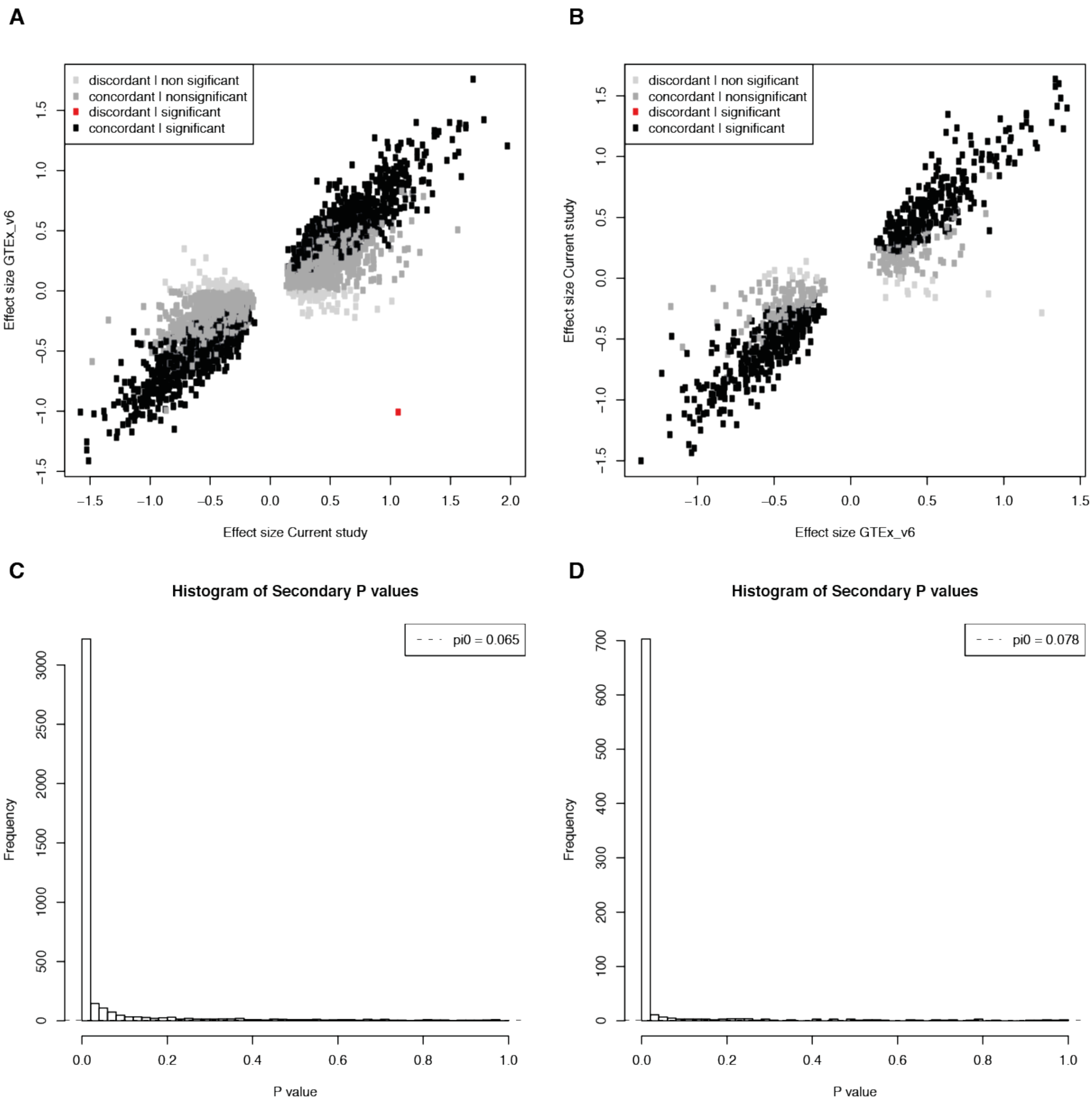

Supplemental Figure 4:*Comparison of eQTL results with GTEx*. We obtained P-values and effect size estimates from the GTEx eQTL results for significant eQTLs (P < 1e-5) of the current study that were also analysed in GTEx **a-b)** and vice versa **c, d)**. Scatterplots **a, c)** show the effect size estimates. Histograms show the distributions of eQTL P-values **b, d)** and estimates pi_0 of the proportion of true null hypothesis estimated by the q-value method.

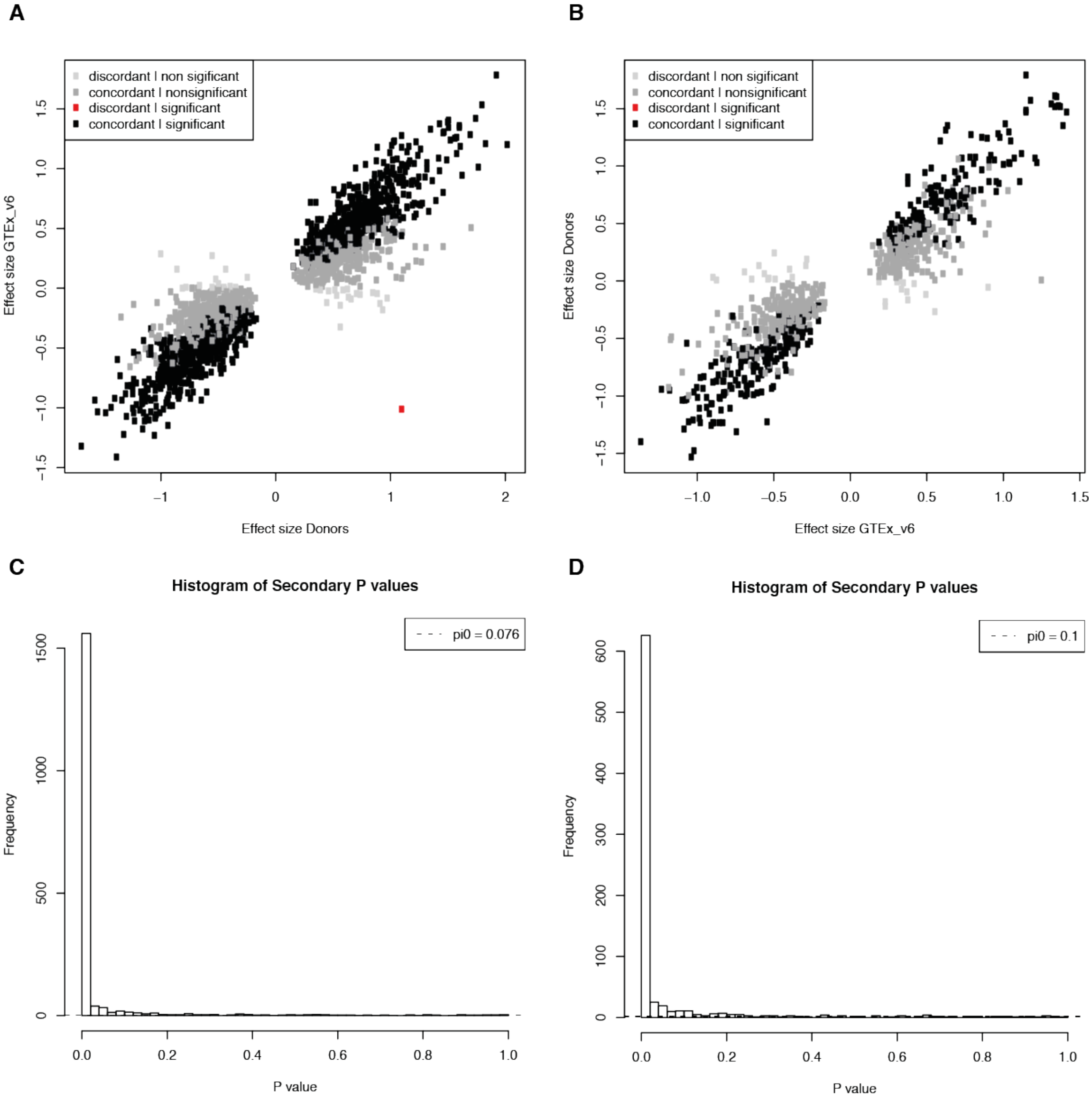

Supplemental Figure 5:*Comparison of eQTL results obtained from donor samples only with GTEx*. We obtained P-values and effect size estimates from the GTEx eQTL results for significant eQTLs (P < 1e-5) of the current study that were also analysed in GTEx **a-b)** and vice versa **c, d)**. Scatterplots **a, c)** show the effect size estimates. Histograms show the distributions of eQTL P-values **b, d)** and estimates pi_0 of the proportion of true null hypothesis estimated by the q-value method.

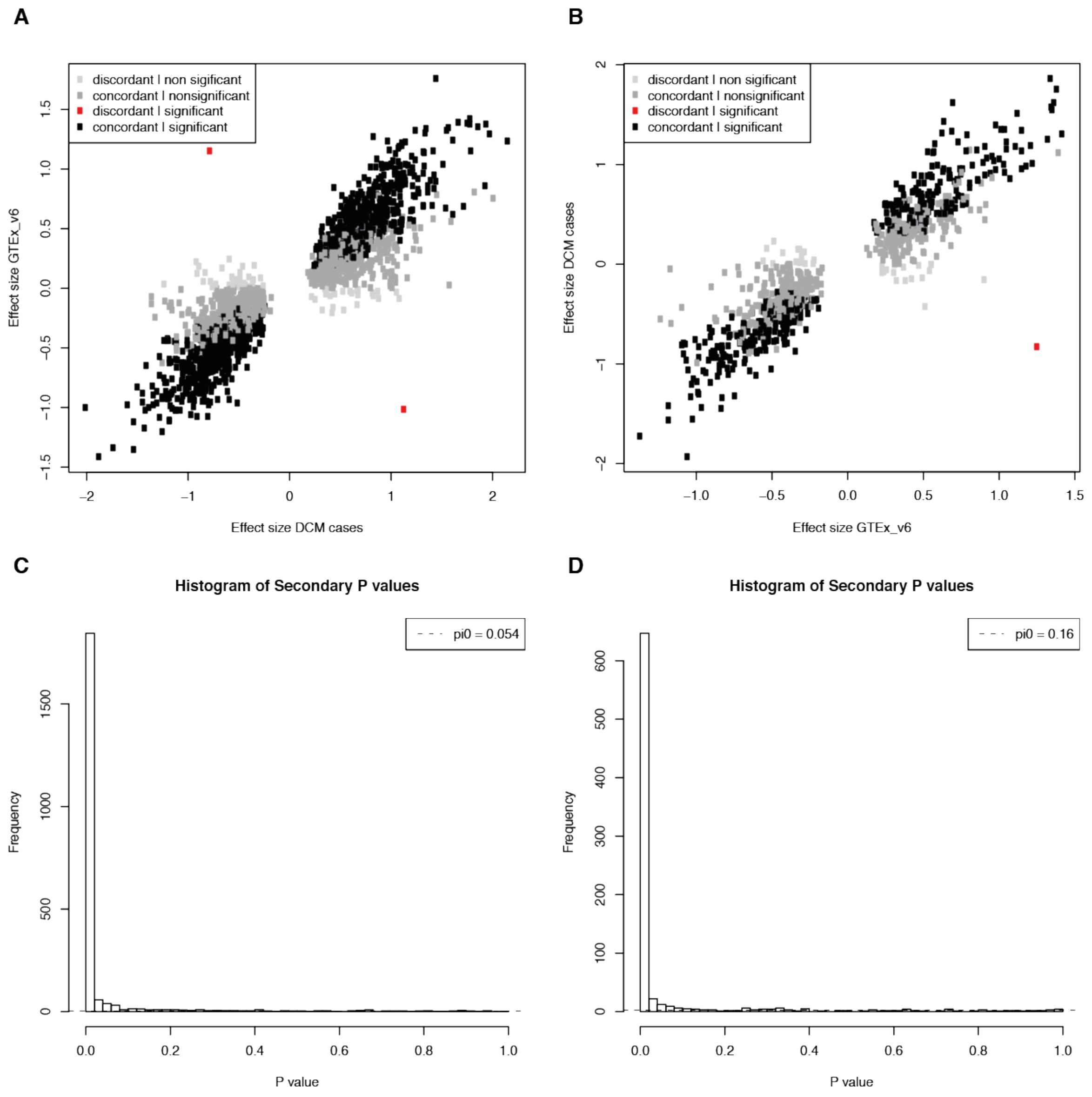

Supplemental Figure 6:*Comparison of eQTL results obtained from DCM patients only with GTEx*. We obtained P-values and effect size estimates from the GTEx eQTL results for significant eQTLs (P < 1e-5) of the current study that were also analysed in GTEx **a-b)** and vice versa **c, d)**. Scatterplots **a, c)** show the effect size estimates. Histograms show the distributions of eQTL P-values **b, d)** and estimates pi_0 of the proportion of true null hypothesis estimated by the q-value method.

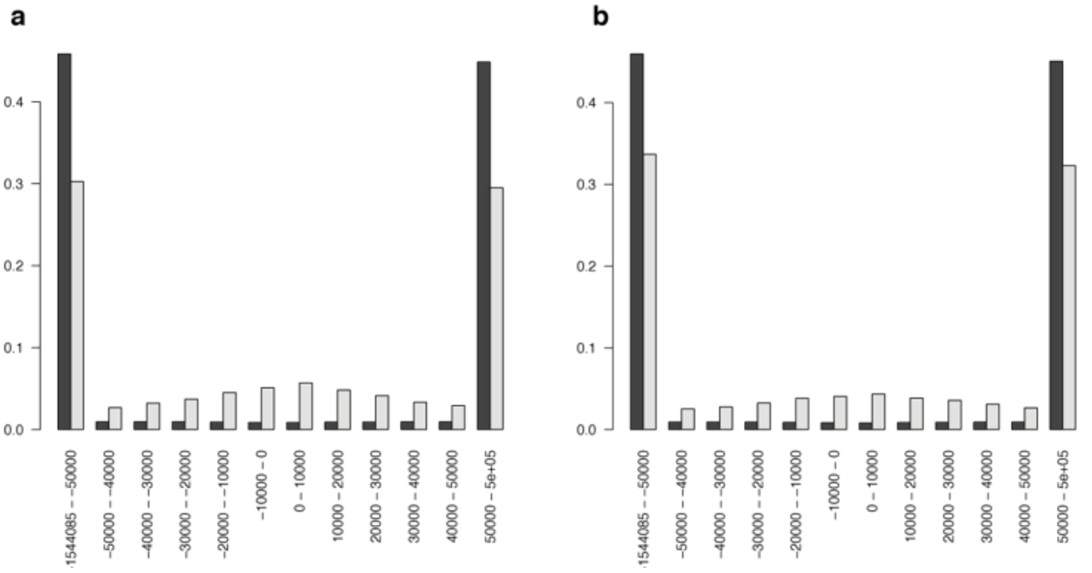

Supplemental Figure 7: *Distance of QTL SNPs to target genes and exons*. Histograms show the distribution of SNPs with QTL (light grey) and without QTL (dark grey) for **a)** sQTLs and **b)** eQTLs.

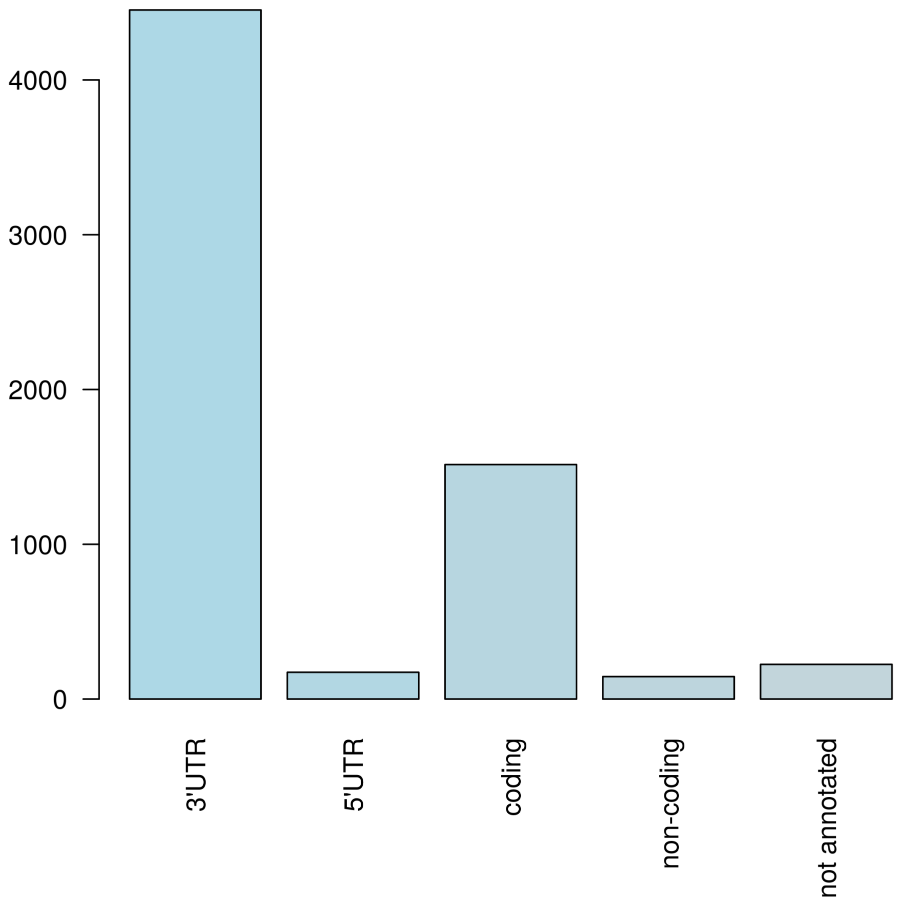

Supplemental Figure 8: *Functional annotation of all SNPs used in the ASE analysis*. All 6509 SNPs that are heterozygous in at least one individual and detected at high quality on both the genotyping microarray and in the RNA-seq variant calling were classified based on their relative location in their respective gene. There is a clear bias towards 3'UTR, likely caused by increased sequencing depth due to poly A selection for the RNA-seq analysis. Legend: 3'UTR = 3' untranslated region, 5'UTR = 5' untranslated region, coding = protein-coding exon, non-coding = non-protein-coding region (lncRNA, miRNA, pseudogenes), not annotated = no gene annotation available, i.e. unknown gene region.

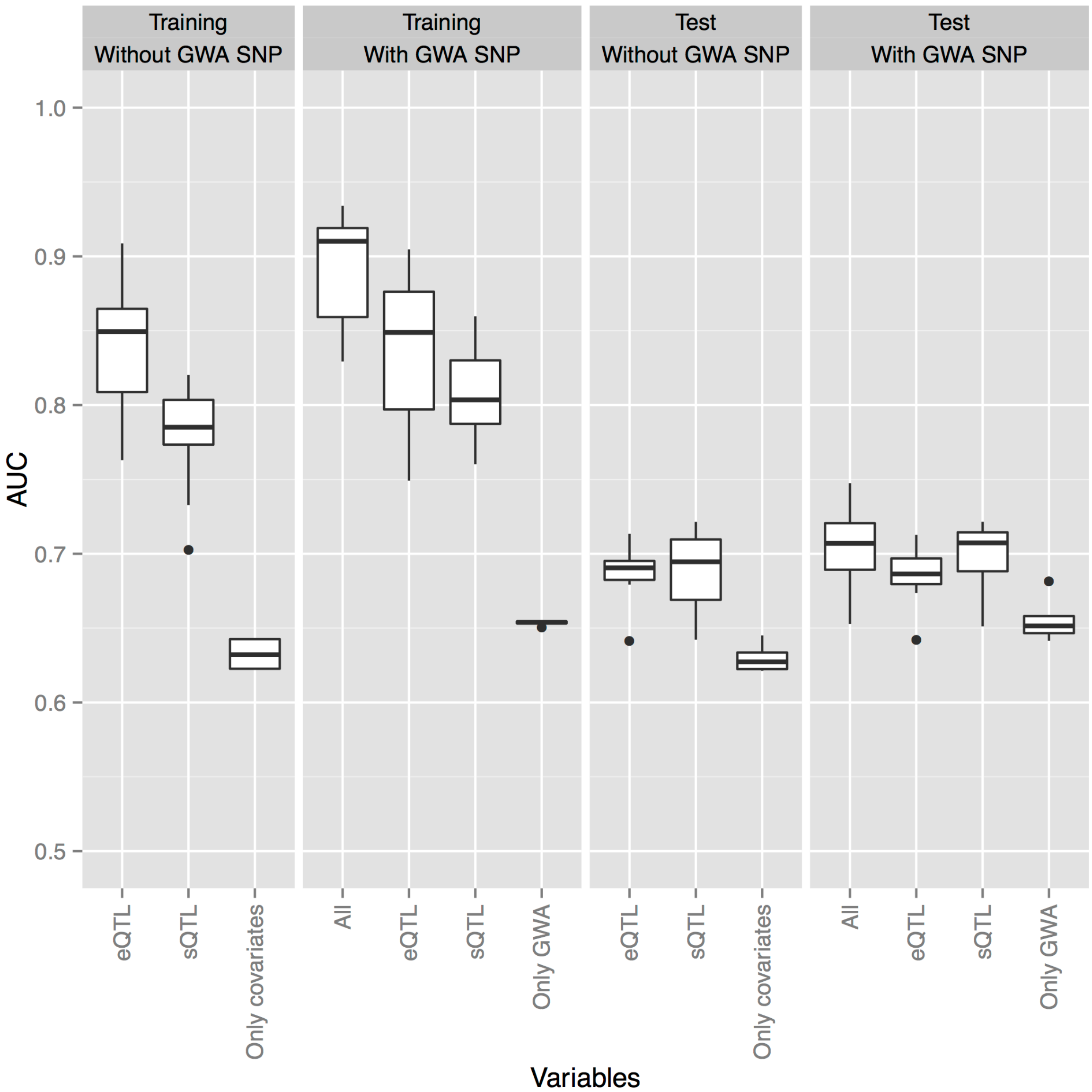

Supplemental Figure 9: *Evaluation of a multilocus DCM risk score*. The x-axis shows the different sets of candidate variables used for model training (eQTL: all SNPs with eQTL ($P < 1e-5$), sQTL: all SNPs with sQTL ($FDR < 0.05$), GWA: rs9262636). All sets contain the covariates age and sex. The y-axis shows the area under the receiver operator characteristics curve (AUC). Boxplots indicate the distribution of AUC values over the ten cross validation runs.

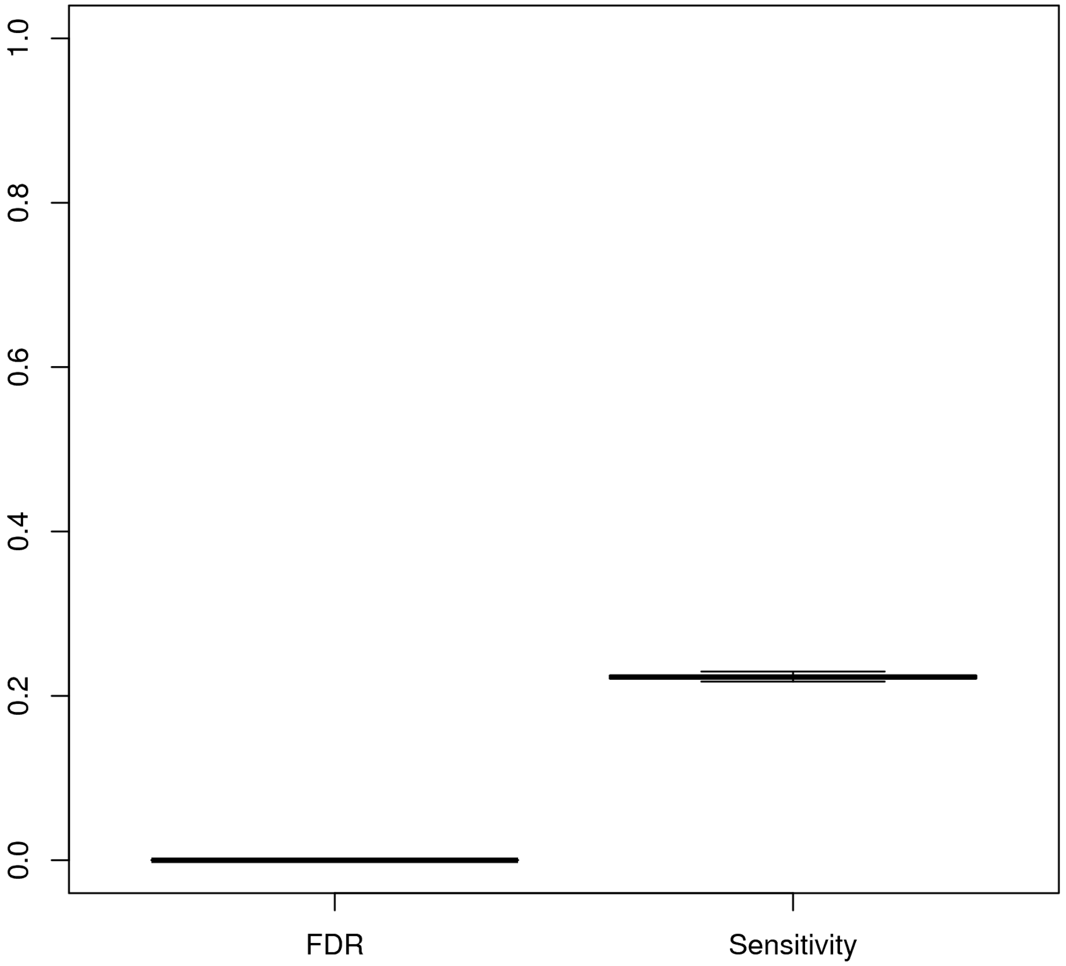

Supplemental Figure 10: *Evaluation of differential splicing detection by simulations*. The boxplots show the distribution of performance measures in 100 repeated simulation runs. The predictions were compared to the simulated differential exons to compute the sensitivity (TP / (FN + TP)) and false discovery rate (FP / (FP + TP)).

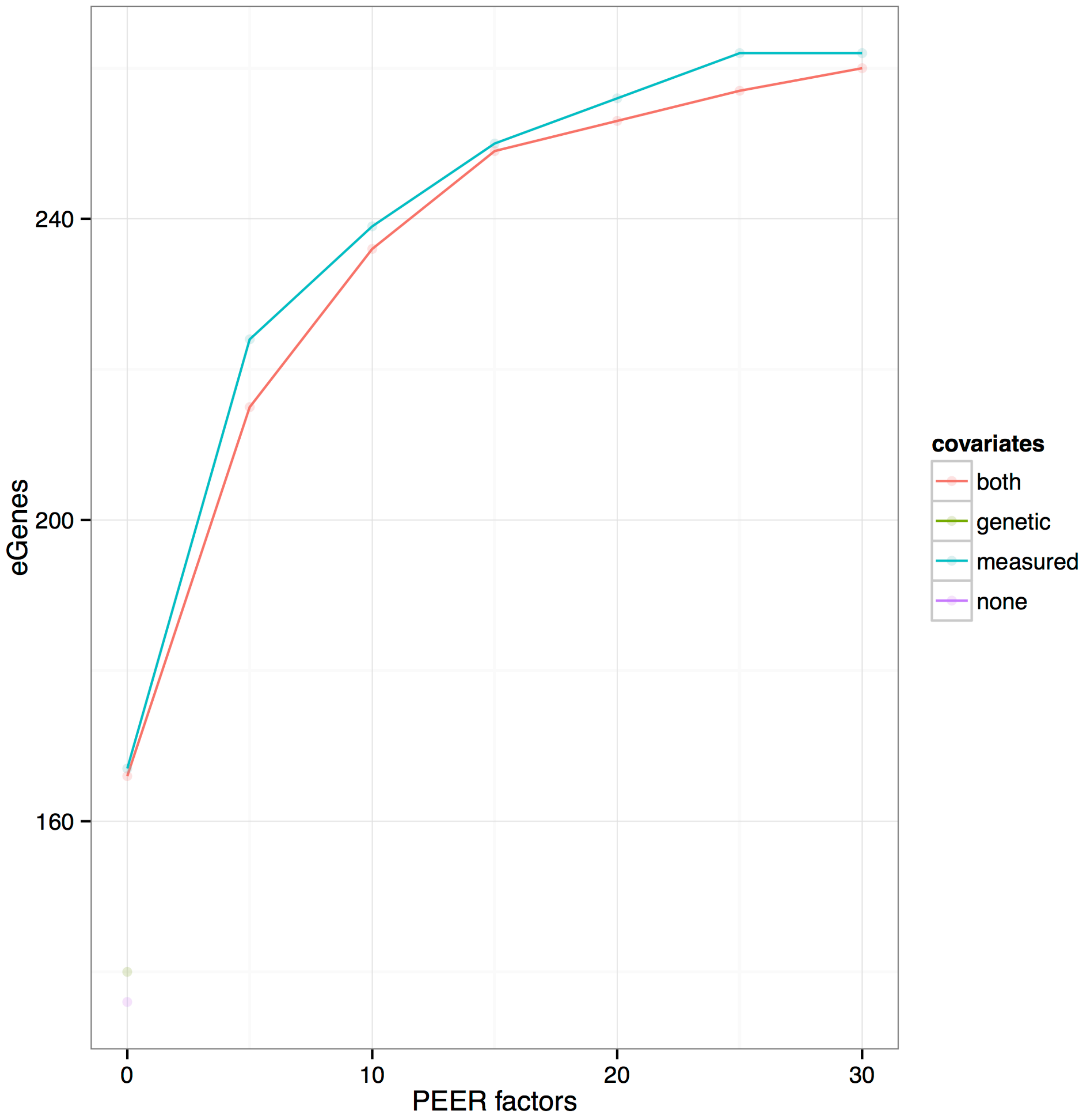

Supplemental Figure 11: *Effect of confounding factors on the detection of cis eQTLs*. The number of genes with significant *cis* eQTLs (eGenes) on chromosome 20 is shown for different combinations of covariates with inferred PEER factors. Covariates in the model are: none (purple), measured clinical covariates (cyan), the first 3 principle components of the genotype data (green) or both clinical and genetic covariates (red).

Supplemental Tables

Supplemental Table 1: *Sample selection based on quality control criteria.* For details on the selection criteria see Methods.

	DCM	Donors
Initial population	128	129
Low RNA quality removed	0	21
Non-Europeans removed	31	0
Used for analysis	97	108

Supplemental Table 2: *Summary of the two study populations*. Summary of clinical covariates sex and age by study center.

Status	Site	N	Female	Male	Avg. age	sd(age)
control	Australia	20	10	10	32.2	11.6
	Hungary	49	21	28	45.6	11.9
	Miami	4	1	3	53.5	28.9
	Nashville	35	13	22	38.5	15.5
	Σ	108	45	63	41.1	14.8
DCM	London	97	15	82	42.3	12.8

Supplemental Table 3: *Summary clinical parameters of the DCM patients.*

**No of patients**	97
**Reason for surgery, No**	
Transplant	49
LVAD implantation	48
**Family history, No**	9
**Age, mean (SD)**	
At diagnosis	39 (13)
At surgery	42 (13)
**LVEF, %, mean (SD)**	22 (9)
**FS, %, mean (SD)**	10 (4)
**LVEDD, mm, mean (SD)**	70 (12)

Supplemental Table 4: *Summary statistics of the RNA-seq data set.*

(in Mio. reads)	Group	Min.	1st Qu.	Median	Mean	3rd Qu.	Max.
**Mapped**	Donor	106.8	168.2	183.2	184	202.3	231.9
	DCM	90.5	163.3	174.9	172.7	187.9	246
**Paired in sequencing**	Donor	106.8	168.2	183.2	184	202.3	231.9
	DCM	90.5	163.3	174.9	172.7	187.9	246
**read1**	Donor	53.67	83.39	90.56	91.39	101.5	117.4
	DCM	50.69	82.01	88.1	87.35	94.34	123.4
**read2**	Donor	53.14	83.95	93.3	92.58	101.6	114.5
	DCM	39.81	80.62	85.83	85.37	93.07	122.6
**Properly paired**	Donor	90.65	134.8	145.8	147.9	163.2	193.8
	DCM	64.49	131.3	140.3	137.6	150.9	188.4
**With itself and mate mapped**	Donor	104	156	169.7	172	191.8	222.7
	DCM	77.28	155.7	166.1	165.2	180.8	236.3
**Singletons**	Donor	2.779	6.551	8.606	11.99	18.8	26.71
	DCM	4.668	6.161	7.122	7.545	8.147	17.43
**RIN score**	Donor	6.5	7.575	8.3	8.198	8.8	10
	DCM	6.3	7.3	7.6	7.586	7.9	8.7

Supplemental Table 5: *Characteristics of the RNA-seq data set*. Table shows for each sample: the total number of RNA-seq reads, mapped reads, paired reads, number of reads of each end of the paired end sequencing (read1, read2), number of reads that are properly paired after alignment, number of reads which also have their mate read mapped and the RNA integrity number (RIN score).

Sample	Total reads	Mapped reads	Paired reads	Read1	Read2	Properly paired	Read and mate read mapped	Singletons	RIN score
20AH01746	157643232	157643232	157643232	80524426	77118806	126576560	148798216	8845016	7.6
20AP01457	191791743	191791743	191791743	96307732	95484011	156865710	184669350	7122393	8.1
20AR01418	167985357	167985357	167985357	84329710	83655647	133218948	161612052	6373305	8.2
20BB01870	174868904	174868904	174868904	88320081	86548823	142934686	167789152	7079752	7.7
20BM01499	167019581	167019581	167019581	83785172	83234409	136605728	161293470	5726111	7.6
20CG01120	177016411	177016411	177016411	91183798	85832613	142948746	166126834	10889577	6.3
20CH01353	178954910	178954910	178954910	89877108	89077802	146508630	171754344	7200566	8.2
20CP01865	124199353	124199353	124199353	69198672	55000681	87276270	106767926	17431427	7.6
20CS01710	168810789	168810789	168810789	84740297	84070492	135605806	162830740	5980049	8.3
20CT01836	171079386	171079386	171079386	85748396	85330990	134472574	165210996	5868390	8.2
20DB01398	168032170	168032170	168032170	85588474	82443696	135681056	158878652	9153518	7.3
20DH01138	186326868	186326868	186326868	94005252	92321616	151220886	178965056	7361812	8
20DH01701	197690440	197690440	197690440	99196462	98493978	160031592	191033456	6656984	7.9
20DL01300	134654301	134654301	134654301	68779989	65874312	105331792	127279848	7374453	7.1
20DS01225	195578792	195578792	195578792	98761762	96817030	155880548	188000226	7578566	7.6
20DW01830	179267544	179267544	179267544	89805661	89461883	143759784	173281668	5985876	8.1
20EC01205	186707021	186707021	186707021	93881433	92825588	146779926	179383206	7323815	8
20EG01127	191909660	191909660	191909660	96601562	95308098	150243644	184133118	7776542	8.5
20EL01751	245984537	245984537	245984537	123403082	122581455	188373502	236329004	9655533	7.1
20ES01406	215345252	215345252	215345252	107064139	108281113	169592030	204825268	10519984	8
20EZ01759	128226406	128226406	128226406	65280228	62946178	102699332	121217062	7009344	7.1
20GC01665	186445286	186445286	186445286	93379696	93065590	152990862	180578954	5866332	6.4
20GF01948	199830258	199830258	199830258	100760914	99069344	158973596	191771430	8058828	7.1
20GJ01209	190205311	190205311	190205311	95717596	94487715	145185616	182373260	7832051	7.9
20GM01331	172245683	172245683	172245683	86584994	85660689	141897226	165146956	7098727	7.1
20HW01332	170541949	170541949	170541949	86074830	84467119	134311902	164039770	6502179	7.5
20IS01302	172532315	172532315	172532315	87108579	85423736	137629504	165858638	6673677	7
20JB01501	175402963	175402963	175402963	87978714	87424249	142310772	169169806	6233157	8
20JC01456	172304724	172304724	172304724	86599832	85704892	139160342	165683344	6621380	7.8
20JC01720	164795115	164795115	164795115	84171889	80623226	132016724	155675132	9119983	7.3
20JD01274	167533916	167533916	167533916	84232555	83301361	116613116	160239982	7293934	7.6
20JD01577	179834657	179834657	179834657	90200490	89634167	144328994	173213176	6621481	8.1

Sample	Total reads	Mapped reads	Paired reads	Read1	Read2	Properly paired	Read and mate read mapped	Singletons	RIN score
20JG01849	173810606	173810606	173810606	87074484	86736122	140352640	168065416	5745190	7.5
20JH01781	196189759	196189759	196189759	98256152	97933607	158182602	189880786	6308973	7.5
20JH01887	200558313	200558313	200558313	101137497	99420816	158855108	192411166	8147147	7.4
20JM01263	179993914	179993914	179993914	90997854	88996060	143670140	172869020	7124894	7.8
20JM01785	182769727	182769727	182769727	92685323	90084404	150904060	174529786	8239941	7.5
20JP01292	170412553	170412553	170412553	88096074	82316479	135915466	159167392	11245161	6.7
20JR01203	176369680	176369680	176369680	90873581	85496099	136361016	165395360	10974320	7
20JT01288	178594349	178594349	178594349	89749855	88844494	144059904	171030046	7564303	7.4
20JW01850	216689934	216689934	216689934	109275263	107414671	172753790	208495932	8194002	7.4
20KM01480	202647510	202647510	202647510	101710291	100937219	163943930	194955058	7692452	7.8
20LB01727	150437392	150437392	150437392	75569600	74867792	120821686	145099680	5337712	7.3
20LD01214	156555268	156555268	156555268	78946447	77608821	124545852	150477976	6077292	8.3
20LH01824	145068059	145068059	145068059	73964139	71103920	116051326	136894514	8173545	7.5
20LO01967	160838180	160838180	160838180	82827639	78010541	129707592	150896378	9941802	8.4
20MC01828	161029705	161029705	161029705	80864703	80165002	128690630	155131158	5898547	8.6
20MC01968	174528630	174528630	174528630	88698635	85829995	139141434	166043188	8485442	6.6
20MD01251	129540240	129540240	129540240	65893170	63647070	101966664	121286018	8254222	7.4
20MF01454	177636072	177636072	177636072	89562919	88073153	136882210	170258946	7377126	7
20MH01845	175144015	175144015	175144015	87935607	87208408	140326910	168796582	6347433	7.8
20MH01879	169497141	169497141	169497141	84910043	84587098	138526598	164060784	5436357	7.7
20ML01902	170492409	170492409	170492409	85622249	84870160	134194882	165237000	5255409	7.7
20MS01733	186401237	186401237	186401237	93598329	92802908	148735938	180808938	5592299	7.8
20MT01164	195597610	195597610	195597610	98267597	97330013	158647406	187387094	8210516	6.6
20NB01855	126920617	126920617	126920617	69926526	56994091	92389314	110718222	16202395	7.4
20OH01215	178082209	178082209	178082209	89465875	88616334	141751052	170616592	7465617	7.5
20PC01455	161000846	161000846	161000846	80788239	80212607	124512706	154454824	6546022	7.1
20PD01505	169179571	169179571	169179571	84817377	84362194	134936820	162927844	6251727	6.5
20PF01735	163653876	163653876	163653876	82176350	81477526	131559466	157946152	5707724	8.6
20PH01298	164675618	164675618	164675618	82706826	81968792	132727060	157901546	6774072	7.3
20PL01827	211585922	211585922	211585922	106211723	105374199	172138918	205324284	6261638	7.7
20PM01841	187918349	187918349	187918349	94336845	93581504	153878404	182277742	5640607	7.9
20PP01831	128784054	128784054	128784054	64581776	64202278	102600462	124115564	4668490	6.7
20PR01392	196483074	196483074	196483074	98570589	97912485	158850032	188640634	7842440	7.9
20PS01694	111226073	111226073	111226073	61098771	50127302	79441730	97202938	14023135	7.9
20PW01714	160464655	160464655	160464655	80517957	79946698	131273960	154912498	5552157	7.9
20RB01952	90498182	90498182	90498182	50689744	39808438	64491760	77280140	13218042	7.4
20RC01659	145767697	145767697	145767697	73051329	72716368	111687700	140569158	5198539	7.4
20RC01868	135227258	135227258	135227258	68558981	66668277	104810232	129066582	6160676	6.8
20RD01346	191311590	191311590	191311590	96281006	95030584	147742928	183465320	7846270	8.4
20RE01817	155433383	155433383	155433383	79216883	76216500	125495634	147544912	7888471	7.4
20RH01884	167591408	167591408	167591408	85152959	82438449	133643316	159504340	8087068	7.4

Sample	Total reads	Mapped reads	Paired reads	Read1	Read2	Properly paired	Read and mate read mapped	Singletons	RIN score
20RJ01217	179794079	179794079	179794079	90791638	89002441	143931754	172918712	6875367	7.5
20RJ01812	163476045	163476045	163476045	81958827	81517218	129162870	157919108	5556937	7.9
20RL01528	189430969	189430969	189430969	95067865	94363104	154844790	182893442	6537527	7.8
20RP01386	190159011	190159011	190159011	95902134	94256877	153746580	182802816	7356195	8.1
20RP01833	176382452	176382452	176382452	88597962	87784490	143660706	171123596	5258856	8.1
20RS01126	185723099	185723099	185723099	93525494	92197605	154628364	179863642	5859457	6.9
20RT01367	194868463	194868463	194868463	97822626	97045837	158368176	187530410	7338053	7.6
20RV01241	194784350	194784350	194784350	98012945	96771405	158676452	188445834	6338516	7.9
20RW01403	190074513	190074513	190074513	95915037	94159476	149880086	182428676	7645837	7.7
20RW01823	189498928	189498928	189498928	96835431	92663497	150511212	178774446	10724482	8
20SB01532	170223713	170223713	170223713	85457330	84766383	135313570	164034516	6189197	8.7
20SC01497	152771498	152771498	152771498	76620553	76150945	118732566	146955922	5815576	6.9
20SD01603	185330758	185330758	185330758	92957008	92373750	151887830	178775778	6554980	7.7
20SD01837	163253539	163253539	163253539	82012940	81240599	131817618	157300490	5953049	7.9
20SE01776	152996870	152996870	152996870	77928595	75068275	123615460	144631362	8365508	7
20SF01123	187020087	187020087	187020087	94218774	92801313	150232568	180194276	6825811	7.8
20SH01483	196122248	196122248	196122248	98358488	97763760	159588768	189215094	6907154	7.7
20SI01410	135428800	135428800	135428800	68118937	67309863	108252478	130571570	4857230	8
20SP01339	159941841	159941841	159941841	80433925	79507916	131369164	154759624	5182217	8
20SW01296	185876783	185876783	185876783	93553637	92323146	144708110	178238384	7638399	8
20TH01341	169514270	169514270	169514270	85339134	84175136	137933734	162162798	7351472	6.5
20TU01919	220341511	220341511	220341511	111128605	109212906	175115844	212071184	8270327	7.1
20TW01940	106409328	106409328	106409328	58844412	47564916	76611824	92316724	14092604	7.4
20WC01228	177255825	177255825	177255825	90136315	87119510	143248554	168702642	8553183	7.4
CON_573MB	188346372	188346372	188346372	95236317	93110055	159140388	180842380	7503992	7.6
CON_N0762	186170619	186170619	186170619	94096774	92073845	156485076	179024238	7146381	8.9
CON_N1212	168950913	168950913	168950913	77450001	91500912	125889880	148290556	20660357	8
CON_N1754	170592687	170592687	170592687	86390169	84202518	146810690	163516420	7076267	6.7
CON_N1877	193857995	193857995	193857995	97943717	95914278	163150130	186894016	6963979	6.8
CON_N2658	151315293	151315293	151315293	76114705	75200588	128088146	147107788	4207505	8.5
CON_N2765	181763594	181763594	181763594	91456276	90307318	153431628	176431236	5332358	8.6
CON_N2917	181244331	181244331	181244331	91154076	90090255	151277488	175892458	5351873	8.5
CON_N3060	192415201	192415201	192415201	97372263	95042938	162290476	185330638	7084563	8.3
CON_N3064	106805865	106805865	106805865	53666915	53138950	90648978	104027064	2778801	6.8
CON_N3077	208529308	208529308	208529308	105500342	103028966	174772588	200104640	8424668	8.2
CON_N3145	192220064	192220064	192220064	97997343	94222721	156527256	180123730	12096334	7.4
CON_N3683	195731390	195731390	195731390	98998814	96732576	163425174	188750124	6981266	8.2
CON_N3736	191220571	191220571	191220571	96734755	94485816	157482322	184100058	7120513	8.3
CON_N3878	185366142	185366142	185366142	94243217	91122925	150932546	176689264	8676878	8.9
CON_N4856	212708991	212708991	212708991	107549243	105159748	180349820	204306902	8402089	7.7
CON_N5288	170677602	170677602	170677602	86303527	84374075	145745272	163989956	6687646	6.5

Sample	Total reads	Mapped reads	Paired reads	Read1	Read2	Properly paired	Read and mate read mapped	Singletons	RIN score
CON_N5713	221456360	221456360	221456360	111972170	109484190	187944032	212921308	8535052	7.5
CON_N7990	181892765	181892765	181892765	91687543	90205222	150837348	176287542	5605223	8.4
CON_N8073	146884806	146884806	146884806	73896217	72988589	123006116	142762310	4122496	8
CON_N9303	152976339	152976339	152976339	77052703	75923636	122642922	147829644	5146695	8.9
CON_N9356	162613915	162613915	162613915	81968741	80645174	138484338	157470620	5143295	8.1
CON_N9511	188445104	188445104	188445104	94887967	93557137	161935914	182992360	5452744	7.2
CON__259M	147708367	147708367	147708367	74453615	73254752	123961880	143188742	4519625	7.4
CON__3083	216578226	216578226	216578226	112248954	104329272	169618498	194033078	22545148	8.9
CON__3091	177434912	177434912	177434912	91746000	85688912	137878054	160002996	17431916	8.8
CON__3108	178879493	178879493	178879493	90124413	88755080	142302572	172678072	6201421	10
CON__3112	192365259	192365259	192365259	88676928	103688331	145894040	170562160	21803099	9.7
CON__3160	179770725	179770725	179770725	84830637	94940088	132335284	154514092	25256633	8.6
CON__4043	195585881	195585881	195585881	101421298	94164583	149599902	175361812	20224069	8.6
CON__4062	175885753	175885753	175885753	80519245	95366508	131608534	154904158	20981595	8.1
CON__4184	195865392	195865392	195865392	98679783	97185609	158654990	189291296	6574096	8.1
CON__429M	205871375	205871375	205871375	104121515	101749860	170088438	197702244	8169131	7.3
CON__455M	208643107	208643107	208643107	105058696	103584411	174663080	202375572	6267535	7.5
CON__461M	194386324	194386324	194386324	98405447	95980877	159801626	186497210	7889114	7.9
CON__475M	168678495	168678495	168678495	84957349	83721146	143704220	163656746	5021749	7.1
CON__5033	166709005	166709005	166709005	84263136	82445869	138615614	160330504	6378501	8.7
CON__5126	202136478	202136478	202136478	93306968	108829510	152240514	179425374	22711104	8.3
CON__5138	201030171	201030171	201030171	101221834	99808337	173063352	195553736	5476435	8.3
CON__5140	183808767	183808767	183808767	84785996	99022771	138589518	163225290	20583477	9.5
CON__6008	168213892	168213892	168213892	78826501	89387391	123668744	143490038	24723854	8.5
CON__6028	217679383	217679383	217679383	110693076	106986307	179866798	207413188	10266195	8.1
CON__6092	176908405	176908405	176908405	89347272	87561133	140283484	170598204	6310201	9
CON__643M	206512172	206512172	206512172	104442152	102070020	172007916	198584580	7927592	6.8
CON__669M	158844497	158844497	158844497	79949769	78894728	134712280	154059914	4784583	7.3
CON__7024	185193886	185193886	185193886	85025268	100168618	139817268	162761696	22432190	9
CON__7040	180953678	180953678	180953678	83186404	97767274	134848890	159238680	21714998	9
CON__7054	184482642	184482642	184482642	87100312	97382330	137998910	158415632	26067010	8.8
CON__7060	142106477	142106477	142106477	65976510	76129967	106351256	126770932	15335545	8.9
CON__711M	181607648	181607648	181607648	94088368	87519280	142182202	162856398	18751250	8.3
CON__713M	208714066	208714066	208714066	106099229	102614837	172937456	199165274	9548792	7.5
CON__827M	138483817	138483817	138483817	69992062	68491755	116653374	133120864	5362953	7.3
CON__843M	159042973	159042973	159042973	80059961	78983012	132098370	154293244	4749729	6.6
CON__916M	231861837	231861837	231861837	117370043	114491794	193783348	222657954	9203883	7.5
CON__H73B	200768992	200768992	200768992	101549970	99219022	170120952	193436974	7332018	9
CON___H12	174193677	174193677	174193677	90173471	84020206	139494576	156377402	17816275	6.8
CON___H13	162117814	162117814	162117814	74540736	87577078	121618836	142455416	19662398	8.4
CON___H14	147258329	147258329	147258329	68233547	79024782	111598008	131346296	15912033	7

Sample	Total reads	Mapped reads	Paired reads	Read1	Read2	Properly paired	Read and mate read mapped	Singletons	RIN score
CON___H20	168245443	168245443	168245443	85793293	82452150	139232456	157422100	10823343	6.6
CON___H22	224657141	224657141	224657141	114625290	110031851	184287914	210242316	14414825	7.1
CON___H24	208963895	208963895	208963895	106648098	102315797	172226312	195552794	13411101	7.8
CON___H25	185809211	185809211	185809211	94769684	91039527	151063480	173663664	12145547	8.1
CON___H26	167006679	167006679	167006679	84101255	82905424	146363448	162269748	4736931	7.1
CON___H27	182676401	182676401	182676401	92379468	90296933	152928932	176195170	6481231	8.2
CON___H28	190948945	190948945	190948945	97464398	93484547	155774092	178530186	12418759	8
CON___H29	188291366	188291366	188291366	86047090	102244276	142686664	165411964	22879402	8.3
CON___H30	157441594	157441594	157441594	79556704	77884890	126123048	147297618	10143976	7.1
CON___H31	167027485	167027485	167027485	84071767	82955718	144493188	162530208	4497277	6.7
CON___H35	191471523	191471523	191471523	87714965	103756558	143172178	168713656	22757867	7.6
CON___H36	177163669	177163669	177163669	86599861	90563808	137618076	166111224	11052445	8.5
CON___H38	227239474	227239474	227239474	114608363	112631111	183953502	219237044	8002430	8.7
CON___H39	204376079	204376079	204376079	99942262	104433817	161341600	191527934	12848145	8.7
CON___H40	205595443	205595443	205595443	106583546	99011897	159089474	184402626	21192817	9
CON___H42	205392775	205392775	205392775	103467813	101924962	169518286	198419408	6973367	8.5
CON___H44	188143436	188143436	188143436	86548240	101595196	142432104	166482410	21661026	8.9
CON___H46	171677780	171677780	171677780	78738803	92938977	128469226	150811092	20866688	8.1
CON___H47	182537179	182537179	182537179	83452439	99084740	132764016	159675828	22861351	8.5
CON___H48	153991381	153991381	153991381	79661877	74329504	118452456	138688764	15302617	8.7
CON___H49	203051355	203051355	203051355	102335713	100715642	164845396	196056910	6994445	8.5
CON___H50	229271024	229271024	229271024	115921676	113349348	192920970	221222034	8048990	8.6
CON___H54	215576844	215576844	215576844	105467815	110109029	174018658	202274682	13302162	8.7
CON___H59	214501689	214501689	214501689	108150516	106351173	177675972	207317210	7184479	9.2
CON___H61	180423698	180423698	180423698	82416380	98007318	135681754	158381160	22042538	8.9
CON___H65	224050419	224050419	224050419	116131753	107918666	174530470	200790272	23260147	9.2
CON___H66	230951154	230951154	230951154	116803751	114147403	180611658	215828512	15122642	9.6
CON___H68	166347409	166347409	166347409	84046551	82300858	138903834	160115974	6231435	8.6
CON___H69	199448940	199448940	199448940	100493367	98955573	162987338	192795844	6653096	8.6
CON___H70	188034445	188034445	188034445	88583858	99450587	138719078	161320648	26713797	9.1
CON___H71	225347465	225347465	225347465	113489834	111857631	184801972	217939002	7408463	9.3
CON___H76	202829901	202829901	202829901	105054866	97775035	155336116	182295694	20534207	8.5
CON___H77	178286435	178286435	178286435	92387119	85899316	135768406	159347650	18938785	8.4
CON___H78	175178226	175178226	175178226	88403343	86774883	137098112	168919782	6258444	9.6
CON___H79	212028472	212028472	212028472	106839749	105188723	172259552	204768990	7259482	9.6
CON___H80	174456531	174456531	174456531	85283211	89173320	135609194	163232230	11224301	8.9
CON___H81	169770791	169770791	169770791	88088011	81682780	128278010	151840732	17930059	9.3
CON___H82	204246163	204246163	204246163	102866181	101379982	165671366	197668648	6577515	8.5
CON___H84	155937321	155937321	155937321	78535477	77401844	127616376	150692382	5244939	9
CON___H85	151251654	151251654	151251654	74021989	77229665	116259548	141815682	9435972	9
CON___H86	151501090	151501090	151501090	69350273	82150817	112228464	133297612	18203478	8.7

Sample	Total reads	Mapped reads	Paired reads	Read1	Read2	Properly paired	Read and mate read mapped	Singletons	RIN score
CON___LV4	212249347	212249347	212249347	103685899	108563448	164249852	198850032	13399315	7.9
CON____10	165224312	165224312	165224312	84143373	81080939	134874444	153342958	11881354	7.8
CON____14	154297575	154297575	154297575	77980365	76317210	129583206	148431980	5865595	7
CON____23	178780234	178780234	178780234	90939069	87841165	147520484	170481524	8298710	7.4
CON____29	187896445	187896445	187896445	95092666	92803779	156315350	180685022	7211423	7.6
CON____H3	162681274	162681274	162681274	74613659	88067615	123422008	143583114	19098160	8.2
CON____H6	169830397	169830397	169830397	85630677	84199720	137375826	164287748	5542649	7.5
CON____H8	153337986	153337986	153337986	72199881	81138105	114448420	131643306	21694680	7.6
CON____H9	134748477	134748477	134748477	63497017	71251460	100777542	115491248	19257229	7.8

Supplemental table 6: *DCM associated genes from Roberts et al. (2015) [20].*

ACTC1	EMD	LDB3	SGCA
ACTN2	EYA4	LMNA	SGCB
ALMS1	FHL2	MYBPC3	SGCD
ANKRD1	FKRP	MYH6	SGCG
APOA1	FKTN	MYH7	SOD2
BAG3	FLT1	NEXN	SYNE1
CAV3	FOXD4	NFKB1	TAZ
CRYAB	FXN	PDLIM3	TBX20
CSRP3	GATAD1	PLEC	TCAP
CTF1	HADHA	PLN	TMPO
DES	HFE	PRDM16	TNNC1
DMD	HOPX	PSEN1	TNNI3
DNAJC19	ILK	PSEN2	TNNT2
DSG2	LAMA2	RBM20	TPM1
DSP	LAMA4	SCN5A	TTN
DTNA	LAMP2	SDHA	VCL

Supplemental table 7: *Genotype accuracy based on variant positions with high coverage (>30 reads) RNA-seq data.*

	DCM	Donors
Accuracy	98.0%	98.9%
Accuracy homozygous	98.9%	99.5%
Non reference accuracy	95.9%	96.9%

Supplemental Table 8: *Summary of the comparison of eQTL results with the GTEx study*. Details for eQTL from the full sample (current study) as well as eQTL from only the DCM patients or only the donor samples are given.

Primary study<br>Secondary Study	Current study<br>GTEx_v6	GTEx_v6<br>Current study	DCM cases<br>GTEx_v6	GTEx_v6<br>DCM cases	Donors<br>GTEx_v6	GTEx_v6<br>Donors
eGenes in primary study	5074	4526	2635	4526	2239	4526
eGenes with SNPs analysed in secondary study	4153	797	2198	797	1822	797
fraction of eGenes with SNPs analysed in secondary study	0.82	0.18	0.83	0.18	0.81	0.18
concordant eQTLs	4042	774	2141	764	1788	770
fraction concordant eQTLs	0.97	0.97	0.97	0.96	0.98	0.97
significant eQTLs in both	1659	577	1204	376	1090	346
fraction significant eQTLs in both	0.40	0.72	0.55	0.47	0.60	0.43
q-value analysis pi_0	0.06	0.08	0.05	0.16	0.08	0.10

Supplemental Table 9: *Gene ontology enrichment analysis results for genes at ASE sites with significant differences between DCM and Donor samples, and with a preferential allele.*

Category	Term	Enrichment p-value
BP	heart development	2.70E-04
	regulation of skeletal muscle cell proliferation	4.00E-04
	system process	1.10E-03
	cardiac cell development	1.35E-03
	muscle structure development	1.51E-03
	tissue development	3.34E-03
	regulation of integrin-mediated signaling pathway	3.91E-03
	negative regulation of MAPK cascade	4.07E-03
	regulation of necroptotic process	4.72E-03
	organelle assembly	4.82E-03
	developmental growth	5.10E-03
	lipid particle organization	5.94E-03
	nucleoside diphosphate metabolic process	6.02E-03
	muscle cell development	6.20E-03
	organic substance catabolic process	6.65E-03
	necrotic cell death	6.98E-03
	mitochondrial fission	7.54E-03
	regulation of blood pressure	7.78E-03
	respiratory gaseous exchange	7.80E-03
	ncRNA 3'-end processing	8.86E-03
	early endosome to late endosome transport	9.11E-03
	actin cytoskeleton organization	9.75E-03
CC	cytoplasmic part	2.10E-04
	intrinsic component of organelle membrane	5.30E-04
	cytoplasm	1.46E-03
	endosomal part	1.54E-03
	extracellular membrane-bounded organelle	1.61E-03
	organelle	1.97E-03
	extracellular organelle	2.07E-03
	vesicle	2.34E-03
	contractile fiber part	2.84E-03
	nuclear membrane part	5.17E-03
	membrane-bounded vesicle	5.22E-03
	actin cytoskeleton	5.49E-03
	contractile fiber	5.78E-03
	endosome	5.87E-03
	endosome membrane	6.33E-03
	cell-cell junction	6.94E-03
	extracellular vesicle	7.41E-03

Supplemental Table 9 continued

Category	Term	Enrichment p-value
MF	protein self-association	4.90E-03
	electron carrier activity	1.01E-02
	MAP kinase phosphatase activity	1.35E-02
	GDP binding	1.55E-02
	solute:cation antiporter activity	1.87E-02
	laminin binding	2.26E-02
	molecular function regulator	2.36E-02
	catalytic activity	2.41E-02
	copper ion binding	2.67E-02
	cytoskeletal protein binding	2.68E-02
	fatty acid binding	3.05E-02
	heme-copper terminal oxidase activity	3.56E-02
	oxidoreductase activity, acting on a heme group of donors	3.88E-02
	organic acid binding	4.08E-02
	enzyme activator activity	4.81E-02
	protein kinase A binding	4.89E-02
	extracellular matrix binding	4.95E-02
	structural constituent of muscle	4.95E-02
